# Decoding NASICON and Its Metal Interface for Solid‐State Batteries

**DOI:** 10.1002/adma.202520270

**Published:** 2026-02-15

**Authors:** Jiaqi Xu, Taiguang Li, Ying Wang, Ruosi Qiao, Quinn Qiao, Yu Chen, Zhou Yu, Chunyi Zhi, Changmin Shi

**Affiliations:** ^1^ Department of Mechanical and Aerospace Engineering Syracuse University Syracuse New York USA; ^2^ Department of Materials Science and Engineering School of Engineering Westlake University Hangzhou Zhejiang China; ^3^ Department of Mechanical and Industrial Engineering Northeastern University Boston Massachusetts USA; ^4^ Department of Mechanical Engineering University of Alabama Tuscaloosa Alabama USA; ^5^ Department of Mechanical Engineering University of Hong Kong Hong Kong China

**Keywords:** design fundamentals and strategies, electrolyte and metal interface, lithium NASICON, sodium NASICON, solid‐state battery

## Abstract

Solid‐state batteries (SSBs) are among the most promising next‐generation energy storage technologies, offering exceptional safety, high energy density, and fast‐charging capability. Among all kinds of solid electrolytes, sodium super ionic conductor (NASICON) is one of the most promising candidates due to its inexpensive material precursors, air stability, and high ionic conductivity. However, it faces a critical challenge: interfacial instability with metal anodes, leading to dendrite formation and penetration that ultimately cause battery failure. Addressing this issue requires a systematic understanding of the solid electrolyte itself, interfacial failure mechanisms, and robust mitigation strategies. Therefore, this review focuses on NASICON as a model system for both sodium‐ and lithium‐based SSBs, providing a comprehensive overview and new insights into NASICON and its metal interfaces. A top‐down approach is adopted, beginning with the fundamentals of NASICON's crystallography, thermodynamics, and kinetics to elucidate its intrinsic properties and interfacial degradation behaviors. Advanced characterization techniques for probing such failures are then reviewed, followed by a comprehensive discussion of mitigation strategies targeting the electrolyte, electrode, and their interface, along with practical insights into NASICON manufacturing. Finally, future research directions are proposed to guide the advancement of NASICON‐based SSBs toward practical commercialization.

## Graphical abstract

This review paper focuses on lithium‐ and sodium‐based NASICON solid electrolytes and their interfaces with metal anodes using a top‐down framework. It begins with NASICON fundamentals, elucidates anode‐induced failure mechanisms, surveys advanced characterization strategies to understand them, summarizes mitigation approaches, and concludes with a forward‐looking perspective on solid‐state batteries enabled by NASICON.

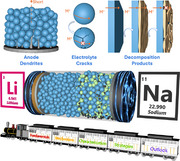

## 1 Introduction

Electrification of the world is becoming one of the defining trends of the 21st century [1, 2, 3, 4, 5, 6]. Energy storage batteries play a critical role in storing electricity that compensates for the electricity supplies generated from solar energy after sunset and from wind power during calm periods [7, 8, 9, 10, 11]. However, currently commercialized battery technologies face severe technical development limitations, particularly safety risks and short driving ranges, primarily due to the use of flammable organic liquid electrolytes and low–energy–density graphite‐based anodes [12, 13, 14, 15]. We acknowledge that conventional liquid‐electrolyte batteries have undoubtedly revolutionized modern life, powering mobile phones, laptops, electric vehicles, drones, and numerous other devices [16, 17, 18]. However, meeting the growing demands for safety, performance, and sustainability will require the development of next‐generation battery cells [19, 20].

To address safety and low‐energy‐density concerns, solid electrolytes paired with metal anodes have emerged as a promising pathway toward achieving a “three‐extreme” battery design: extremely safe, extremely energy‐dense, and extremely fast‐charging [21, 22, 23, 24]. Despite their potential, each class of solid electrolytes faces unique technical challenges. Sulfide and halide solid electrolytes exhibit excellent room‐temperature ionic conductivity (1–10 mS cm^−1^) but lack moisture stability [19, 25, 26, 27]. Polymer electrolytes address the moisture stability issue but typically exhibit low ionic conductivity (10^−7^–10^−4^ S cm^−1^) [28, 29, 30, 31, 32]. Oxide electrolytes offer both moisture stability and moderate to high ionic conductivity (0.1–10 mS cm^−1^), yet they require high‐temperature sintering (e.g., ≥1000°C), which increases production costs [19, 33, 34, 35, 36]. In particular, NASICON‐type oxide electrolytes exhibit superior intrinsic safety due to their high thermal stability and nonflammable nature, which distinguishes them from sulfide‐ and polymer‐based solid electrolytes that are prone to thermal instability and flammability [37, 38].

Among all solid electrolytes, sodium super ionic conductor (NASICON), a member of the oxide electrolyte family, is one of the most promising candidates that stands out, as its precursor materials are relatively inexpensive, making it a viable option for scalable solid‐state battery (SSB) applications [39, 40, 41, 42]. At room temperature, NASICON (e.g., Li_1+x_Al_x_Ti_2‐x_ (PO_4_)_3 (_LATP), Li_1+x_Al_x_Ge_2‐x_ (PO_4_)_3 (_LAGP), Na_3_Zr_2_Si_2_PO_12 (_NZSP)) typically exhibits an ionic conductivity of approximately 10^−4^–10^−3^ S cm^−1^ [43, 44, 45]. Their oxidation stability ranges from approximately 3.5–5 V vs. Li^+^/Li for Li‐based NASICONs, and 3.4–6 V vs. Na^+^/Na for Na‐based NASICONs, depending on the specific composition and dopants [46, 47, 48, 49]. NASICON exists in both Na‐based and Li‐based forms, conducting Na^+^ and Li^+^ ions, respectively. Regardless of the type, NASICON suffers from a critical challenge: chemical reduction at the metal anode interface, leading to battery cell shorting failure. While numerous studies have explored synthesis and interfacial engineering strategies to mitigate this instability, these approaches still lack a universal and rational design framework [42, 50, 51].

A fundamental understanding of NASICON's crystallography, thermodynamics, and kinetics is essential not only for comprehending the intrinsic properties of NASICON materials but also equally important for uncovering the mechanisms of interfacial degradation and failure [52]. Such insights are the foundation for developing visualization approaches that capture these failure processes and for designing targeted engineering strategies to overcome the associated interfacial challenges. In parallel, while understanding NASICON fundamentals and mitigating its instability with metal anodes, it is equally important to focus on practical manufacturing considerations to enable scalable, reliable applications.

**FIGURE 1:**
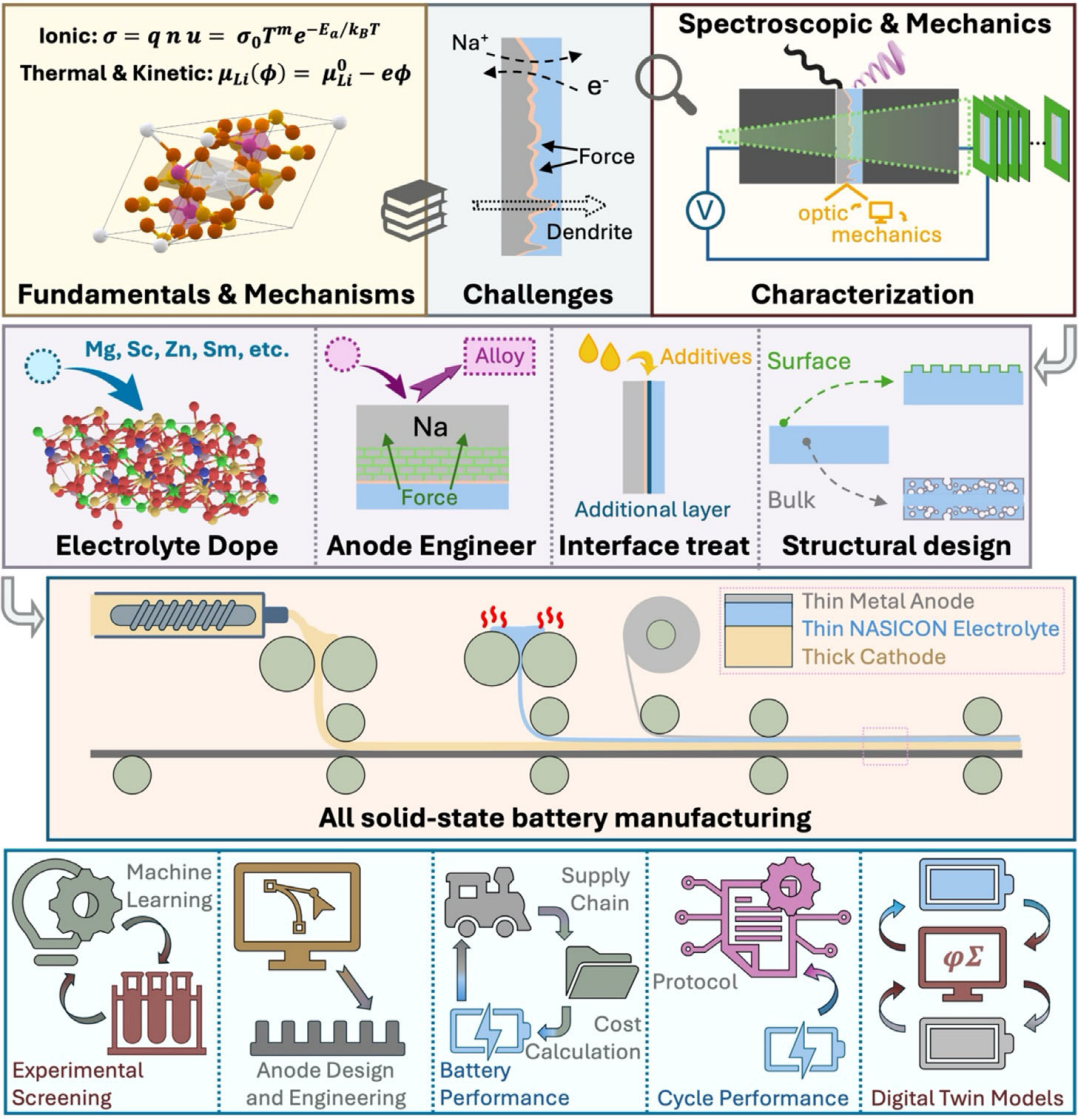
Overview of the top‐down review on NASICON and its metal interface.

In this review, we adopt a top‐down approach to decode both Na‐ and Li‐based NASICON materials and their interfaces with metal anodes (Figure 1), concluding the technical sections with a focus on manufacturing perspectives. We begin with the crystallographic framework and ion‐conduction mechanisms, followed by thermodynamic and kinetic analyses of interfacial instability. Based on theoretical analysis, we discuss the failure mechanism from the experimental side. After that, we bridge theory with experimental observations, detailing practical characterization techniques for interfacial degradation. Next, we provide a detailed review of state‐of‐the‐art strategies to suppress metal dendrite penetration, covering electrolyte, electrode, and interface engineering. As important as lab‐scale research, we also provide practical manufacturing insights to enable NASICON commercialized applications. Finally, based on the authors’ best knowledge and vision, we provide our perspective on the promising research directions and critical innovation needs for advancing NASICON‐based solid‐state batteries toward commercialization.

## 2 NASICON Fundamentals

### 2.1 Fundamentals of Compositions and Crystal Structures

The NASICON family has the general chemical formula AMM′ (XO_4_)_3_, where A is typically Na^+^/Li^+^, M and M′ sites are usually occupied by tetravalent or trivalent transition metal cations (e.g., Zr^4+^, Ti^4+^, Sc^3+^, Y^3+^), and the XO_4_ tetrahedra are polyanionic units such as PO_4_^3−^ or SiO_4_^4−^ [53]. In Na‐based NASICONs, polyanion substitution is common, such as replacing P with Si in Na_x_M_2_ (XO_4_)_3_ (X = Si, P), with the Na^+^ concentration adjusted correspondingly to maintain charge neutrality, as in the well‐known NZSP [39, 54]. The same framework also hosts Li‐ion conductors. For example, LATP and LAGP retain the NASICON polyanion backbone while substituting Li^+^ for Na^+^ [33]. In the following, we use Na‐ion NASICONs to illustrate the crystallographic fundamentals of the NAISCON framework.

**FIGURE 2:**
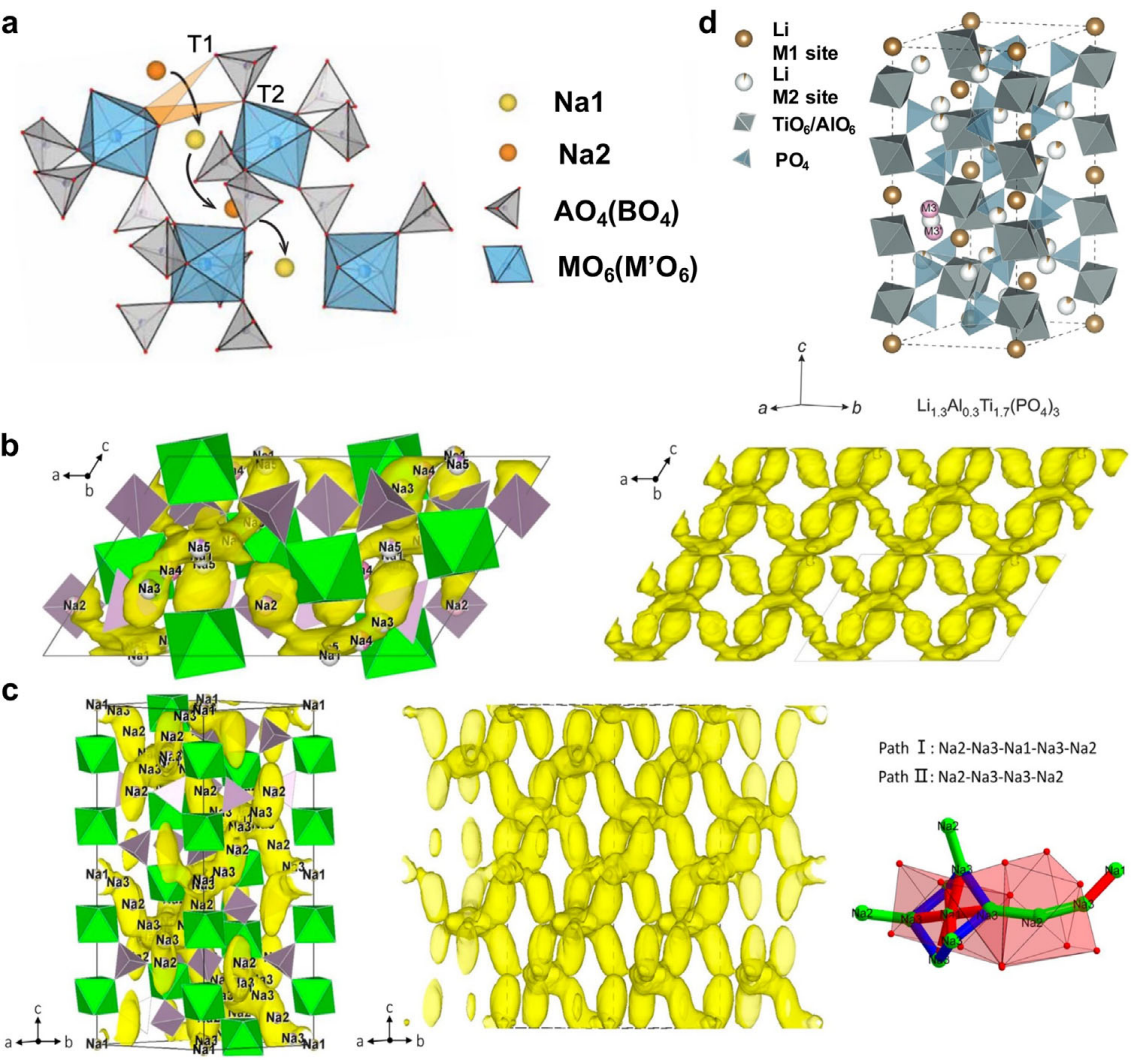
Crystal structure and Na^+^ ion diffusion pathways in rhombohedral and monoclinic NASICON‐type materials. (a) Crystal structure of the rhombohedral R‐3c NASICON, highlighting two bottleneck triangles (T1 and T2). Reprinted with permission from Wang et al. [54] Copyright (2023) Springer Nature. Na^+^ ion diffusion pathways in (b) monoclinic (c) rhombohedral Na_3_Zr_2_Si_2_PO_12_ at 1400 K from AIMD simulations. Green, light purple, and yellow represent ZrO_6_ octahedra, Si (P)O_4_ tetrahedra, and Na atoms, respectively. The Na^+^ ion probability density is shown as yellow isosurfaces. Two possible local pathways are indicated: Path I (Na2‐Na3‐Na1‐Na3‐Na2, blue arrows) and Path II (Na2‐Na3‐Na3‐Na2, green arrows). Reprinted with permission from Zhang et al. [40] Copyright (2019) John Wiley and Sons. (d) Crystal structure of NaSICON‐type rhombohedral LATP, Reprinted with permission from Scheiber et al. [58] Copyright (2024) John Wiley and Sons.

The NASICON framework consists of corner‐sharing MO_6_ octahedra and XO_4_ tetrahedra, creating large interstitial sites and a well‐connected 3D network of migration channels for A‐ions (Figure 2a) [54, 55, 56]. This 3D architecture provides multiple ion pathways and reduces transport bottlenecks compared to lower‐dimensional systems. Structurally, NASICON is commonly described by three phases: C2/c α‐NASICON, typically stable at low temperature close to 300 K, β‐NASICON phases, typically stable at ∼300‐450 K, and the rhombohedral R‐3c γ‐NASICON, the highest symmetry structure, stabilized above 450 K [57]. The *γ* phase features characteristic 3D A‐ion migration channels. However, upon cooling after calcination or with specific substitutions (e.g., adding Na_2_SiO_3_ to adjust the Na/Si content), it transforms into the monoclinic C2/c form, where ion migration is predominantly restricted to two dimensions, thereby limiting transport efficiency [56].

Ion transport bottlenecks along the migration pathways are critical to the ionic conductivity in NASICONs. In rhombohedral R‐3c, the triangular bottlenecks between the A1 and A2 sites, denoted as T1 and T2 in Figure 2a, correlate positively with A‐ion conductivity: larger windows generally lower migration barriers and increase hop frequency [54, 55, 56]. Upon transformation to monoclinic C2/c, the lowered structural symmetry increases distortions in the T1–T2 migration channels. This narrows bottleneck radii and raises migration energy barriers, which reduces A‐ion hopping frequency and overall conductivity.

Moreover, polyanions in NASICON, such as PO_4_^3−^, SiO_4_^4−^, and SO_4_^2−^, are another key factor in ion transportation and can be substituted or doped to tailor framework rigidity, migration pathways, and electrochemical stability [56]. The rigid polyanion units (XO_4_^n−^) form the NASICON backbone, stabilizing the lattice over a wide temperature range by suppressing distortions. Tailoring this framework can modulate migration pathways and improve ionic conductivity. For example, altering the Si/P ratio in NZSP changes the bottleneck sizes along Na^+^ diffusion channels and thus the ionic conductivity [40]. In addition, the XO_4_^n−^ framework improves oxidative stability and broadens the electrochemical window [59].

### 2.2 Ion Conduction in NASICONs

The ionic conductivity, *σ*, in solids following the Arrhenius relationship can be described as the product of the charge (*q*), concentration (*n*), and carrier mobility (*u*), with the temperature dependence

(1) $$\sigma =q\;n\;u=\sigma_{0}T^{m}e^{-E_{a}/k_{B}T}$$

where m is typically −1, *k*_B_ is the Boltzmann constant, *T* is the temperature, and *E_a_* is the characteristic activation energy for ion conduction. *E*_a_ is the sum of the formation energy of mobile defects (*E_f_*) and the migration energy barrier (*E_m_*) along the conduction path. In superionic phases, since the concentration of mobile species is independent of temperature, *E_a_* can be expressed in terms of the migration energy *E_m_*. For temperature‐dependent mobile metal ion concentrations in intrinsic and substitutional metal ion conductors, *E_a_* is equal to *E_m_* + *E_f_*/2.

Three primary factors affect the ionic conductivity of NASICONs: (1) Mobile A^+^ concentration (n), tuned by composition/doping and defect chemistry; (2) Migration energy barriers (*E_m_*), set by bottleneck size between A‐ion sites in migration pathways; and (3) Site occupancy/topology, distribution over A1/A2 sites and vacancy fraction, which controls percolation of pathways and hopping correlations [59].

Ion transport in NASICON is vacancy‐mediated hopping on a 3D network of corner‐sharing P/SiO_4_ tetrahedral and ZrO_6_ octahedral units. In the monoclinic C2/c phase, bond valence energy landscapes (BVEL) place Na5 at the highest energy with the lowest occupation. Nevertheless, ab initio molecular dynamics simulations (AIMD) reveal that Na ions diffuse through all five sublattices and that Na5 serves as a crossover site, facilitating fast and anisotropic 3D diffusion, as illustrated in Figure 2b,c. Triangular O_3_ windows between sites define the bottlenecks and aliovalent dopants (e.g., Mg^2+^, Sc^3+^, Ga^3+^) that enlarge these windows, lower the ion migration barrier, and increase ionic conductivity. Increasing Na content, while maintaining a finite vacancy fraction, strengthens Coulomb repulsion and promotes concerted/correlated multi‐ion motion with lower barriers than isolated single‐ion hops. The role of a “paddle‐wheel” mechanism, anion reorientations directly assisting cation hops, remains debated: some studies report correlations [60], whereas recent analyses argue that large‐angle anion rotations are not the rate‐limiting driver of superionic transport [45, 51, 61, 62]. Similarly, Li^+^ migration in LATP or LAGP proceeds via M1 ‐ M2 ‐ M3 transitions, with Li site energetics and hopping connectivity tunable via aliovalent substitution.

#### 2.2.1 Sodium‐Ion NASICON Conductor

In Na‐rich compositions, the high‐symmetry rhombohedral phase offers multiple, well‐connected migration pathways that facilitate rapid ion transport [54, 55]. Undoped NASICONs typically show moderate bulk or grain boundary ionic conductivity of ∼10^−4^–10^−3^ S cm^−1^ at room temperature, largely limited by insufficient mobile carrier concentrations [59]. By contrast, common dopants, including Mg^2+^, Y^3+^, Al^3+^, and Ga^3+^, are extensively studied and used to partially substitute Zr^4+^ on M‐sites and/or P^5+^ on X‐sites of polyanion units in Na_x_M_2_ (XO_4_)_3_ to optimize carrier concentration, phase stability, and sintering [59]. For example, NZSP is a widely studied NASICON, exhibiting ionic conductivity of up to 6.7 × 10^−4^ S cm^−1^ at room temperature, which is limited by the poorly conducting ZrO_2_ and Na_3_PO_4_ phases formed at grain boundaries. Mg‐doped NZSP, such as Na_3.1_Zr_1.95_Mg_0.05_Si_2_PO_12_, can achieve conductivities of 3.5 × 10^−3^ S cm^−1^ at room temperature, since Mg^2+^ dopants enlarge the T1 triangular bottleneck to ∼6.5 Å^2^, significantly reducing activation energy from 0.29 [63] to 0.25 eV [64].

Aided by the automated synthesis methods and high‐throughput computation, composition maps for NASICONs have identified doping rules that maximize ionic conductivity. Key strategies include tuning Na concentration to ∼3.3 per formula unit and applying cation substitutions to obtain an optimal average M‐site radius of ∼0.72 Å in Na_x_M_2_ (XO_4_)_3_. Dual‐ or multi‐cation substitutions (e.g., Sc^3+^/Ce^4+^ or Ge^4+^/Y^3+^) can further boost conductivity to >5 mS cm^−1^, as reported by Wang et al. and Ouyang et al. [54, 55]. Beyond composition, various sintering techniques can deliver additional gains. Adjusting the sintering scheme (e.g., liquid phase sintering, LPS) can reduce grain boundary resistance and double the room‐temperature conductivity of NASICON.

#### 2.2.2 Lithium‐Ion NASICON Conductor

Lithium NASICONs, most prominently LATP and LAGP, are extensively studied oxide electrolytes for solid‐state batteries. Li ions mainly diffuse through three sites in the NASICON framework, labeled M1 (6b), M2 (18e), and a transient/intermediate M3 site, as illustrated in Figure 2d. The occupations of these sites can be tailored by different doping strategies and by stabilizing either the rhombohedral or the monoclinic framework, thereby modulating the migration barrier and percolation of pathways. Aliovalent cation substitution is an effective strategy for Li^+^ NASICONs. Undoped LiTi_2_ (PO_4_)_3_ shows low room‐temperature ionic conductivity (i.e., ∼2 × 10^−6^ S cm^−1^), whereas Al^3+^ doping introduces excess Li^+^ NASICON and expands the migration bottlenecks, yielding optimized LATP compositions with ionic conductivity up to ∼10^−3^ S cm^−1^ [33]. Partial replacement of Ti^4+^ by Ga^3+^ or Fe^3+^ also leads to LATP‐type compositions, increasing Li^+^ conductivity from 2 × 10^−6^ to 4 × 10^−4 ^S cm^−1^ as reported by Fu et al. [65]. These doping strategies simultaneously increase the carrier concentration and lower the activation energy for the Li^+^ migration in the NASICON framework.

Sintering technology in experimental synthesis is commonly applied to improve the ionic conductivity of Li^+^ NASICONs. Co‐sintering Li^+^ NASICONs with Li‐rich additives, such as LiF [66], LiNO3 [67], and Li_3_PO_4_ [68], or forming glass‐ceramic composites such as Li_2_O–Al_2_O_3_–GeO_2_–P_2_O_5_ [65] in LAGP, can reduce grain boundary resistance and enhance total conductivity. Various other sintering techniques are experimentally verified to be effective, including the cold sintering process (CSP). CSP was recently developed by the Randall group, providing a route to densify ceramics at temperatures below 300°C [69]. Liu et al. reported a cold sintering process and a post‐annealing process. The LATP particles were first densified at 120°C and then annealed at 650°C [70]. The obtained LATP ion conductivity is 8.04 × 10^−5^ S cm^−1^. This low‐temperature densification process avoids Li evaporation and produces nanocrystal‐rich grain boundaries, which improve ionic conductivity.

#### 2.2.3 Comparison Between Na^+^ and Li^+^ Conduction in NASICON

The distinct ionic conduction mechanisms of Na^+^ and Li^+^ in NASICON structures are primarily governed by their preference for coordination environments [71, 72]. Li^+^ is smaller and more polarizing, favoring lower coordination numbers (CNs), typically tetrahedral and octahedral with CN = 4–6. In contrast, the larger Na^+^ ion is stabilized in higher‐coordination sites, typically ranging from 6 to 8 [73]. These preferences influence how each ion migrates within the NASICON lattice. In Li‐based NASICONs (e.g., LATP, LAGP), Li^+^ transport proceeds via site‐to‐site hopping between crystallographically distinct, distorted Li coordination polyhedral within a 3D diffusion network, with migration barriers governed by narrow oxygen bottlenecks and transient interstitial‐like transition states [74]. In contrast, Na‐based NASICONs favor frameworks that accommodate larger Na^+^ ions in higher‐coordination sites, where Na^+^ migrates through connected distorted octahedral or prismatic environments with comparatively larger bottlenecks [75]. Consequently, the diffusion pathways and rate‐limiting mechanisms in Na‐based NASICONs are distinct from those in their Li‐based counterparts. Notably, improved densification from high‐temperature or rapid sintering reduces grain boundary resistance and thereby increases ionic conductivity in NASICON. Samples sintered to higher relative densities exhibit higher total ionic conductivities than poorly densified counterparts due to the suppression of blocking grain boundary phases and improved connectivity. While excessive sintering temperatures may lead to alkali‐ion volatilization, optimized sintering conditions balance densification and compositional stability to maximize conductivity [70, 76]. Overall, the contrast in CN preference between Li^+^ and Na^+^ underscores the importance of tailoring the NASICON framework through dopant selection, site engineering, and optimized sintering strategies that balance densification and compositional stability to maximize ionic conductivity [54, 77].

## 3 Interfacial Stability and Degradation at the NASICON‐Metal Anode Interface

Interfacial stability at the NASICON‐metal anode interface is a critical factor governing the electrochemical performance, safety, and long‐term durability of NASICON‐based solid‐state batteries [78]. Despite their high bulk ionic conductivity, NASICON electrolytes often suffer from chemical, electrochemical, and mechanical degradation when in direct contact with reactive alkali‐metal anodes [78]. These degradation processes arise from coupled thermodynamic driving forces, kinetic limitations, and interfacial microstructural features [79]. In this section, we provide a comprehensive overview of the thermodynamic stability, kinetic processes, and interfacial degradation mechanisms at the NASICON‐metal electrode interface.

### 3.1 Theoretical Modeling of Interfacial Stability and Kinetic Processes

Grand potential phase diagrams are widely used to evaluate the material stability under an imposed chemical potential, including the electrochemical stability of solid electrolytes at Li‐metal interfaces. In this framework, for a chosen Li chemical potential *µ*_Li (_i.e., an open Li reservoir), we can identify the set of equilibrium decomposition products from the grand‐potential diagram. The stability of a given solid electrolyte is then measured by the thermodynamic driving force between the current phase and the identified phase equilibria, quantified as the decomposition reaction energy *E_D_* under the applied voltage *ϕ* [80],

(2) $$\begin{array}{cc} E_{D}\left(\phi \right) & =E\left(phase\;equilibra,\phi \right)-E\left(solid\;electrolyte\right)\\ & \;-\;\Delta n_{Li}\mu_{Li}\left(\phi \right) \end{array}$$

where *E* (phase equilibria, *ϕ*) is the energy of the phase equilibria at the potential *ϕ*, *E* (solid electrolyte) is the energy of the solid electrolyte, and Δn_Li_ is the change in the number of Li from the solid electrolyte composition to the phase equilibria composition during the lithiation or delithiation reaction.

The Li chemical potential *µ*_Li_ can further be expressed as a function of applied electrostatic potential *ϕ*

(3) $$\mu_{Li}\left(\phi \right)=\mu_{Li}^{0}-e\phi$$

where $\mu_{Li}^{0}$ is the chemical potential of Li metal, and the potential *ϕ* is referenced to Li metal. In practice, total energies calculated using Density Functional Theory (DFT) modeling are used to construct grand potential phase diagrams. These calculations can be implemented by computational tools such as the Vienna Ab initio Simulation Package (VASP).

The stability of NASICON electrolytes can be evaluated by the thermodynamic and kinetic processes. While the first‐principles calculations showed that NASICONs are thermodynamically unstable against alkali‐metal electrodes (Zhu et al. [80]), NASICON electrolytes can appear kinetically stabilized. For example, the calculated electrochemical window of Na_3_Zr_2_Si_2_PO_12_ is ∼1.11–3.41 V vs Na^+^/Na, which is lower than the measured 0–5 V electrochemical window reported in experiments. This discrepancy reflects that the stability of the NASICON materials arises from both the inherent thermodynamic property and the kinetic process. The decomposition reaction, although kinetically slow, remains thermodynamically favorable at the applied overpotential and may occur over a prolonged period, leading to degradation of battery performance. The formation of the decomposition interface phase is therefore critical to the stability of the NASICON electrolyte and significantly affects the overall performance of solid‐state batteries.

Suppose the decomposition products at the electrolyte‐electrode interface are stable against the metal's chemical potential and electronically insulating. In that case, they can passivate the interface and suppress further reaction, forming a solid electrolyte interphase (SEI). However, in many lithium‐ion NASICONs, this self‐passivation is ineffective: the reduction of LAGP with Li metal forms electronically conducting lithium‐germanium alloys, and the lithiation of LATP forms a titanate with Ti^3+^ or lower valence, both of which are conductive and thus fail to block electrons.

### 3.2 Chemical / Electrochemical Failure Mechanism

#### 3.2.1 NASICON‐Metal Interfacial Degradation

To avoid ambiguity, we define the key interfacial terms used throughout this section as follows: (1) SEI: This term refers to the passivation layer spontaneously formed via electrochemical and/or chemical reduction of the solid electrolyte upon contact with and during cell cycling with reactive alkali metals (e.g., Li, Na, in our cases); (2) Interphase: A broad term encompassing all modified or newly formed regions at the solid electrolyte/metal interface, including both SEI and artificial layers (e.g., intensionally‐added protective layer, ionic liquid, etc.).

As described in Section 3.1, theoretical evidence suggests that in NASICON‐based solid‐state batteries, the interface stability between the solid electrolyte and the metal electrode is a critical challenge for achieving stable cycling performance, due to the complex physicochemical interactions. These interactions are detrimental if left unmodified, as they give rise to various failure modes that compromise the ionic conductivity, structural integrity, and long‐term cycling stability of the solid‐state battery cells. In this section, we primarily focus on failure mechanisms from experimental evidence. A detailed understanding of these phenomena is essential for diagnosing performance degradation and guiding the rational design of robust electrode–electrolyte interfaces. Specifically, the failure mechanisms at the interface can be mainly categorized into two types: (1) chemical/electrochemical degradation, which involves redox reactions as well as phase heterogeneity and secondary‐phase evolution due to thermodynamic and kinetic driving forces; (2) mechanical failure, which originates from interfacial stress accumulation and mismatch.

Decomposition products at interfaces (referring to SEI formation) have a significant impact on the electrochemical performance and long‐term stability of solid‐state batteries. Here, we focused on the chemical compositions formed intrinsically due to the metal‐electrolyte side reactions. Specifically, the SEI acts as an ionically resistive barrier or an electronically conductive pathway (e.g., the formation of Li_2_TiO_3_). The presence of electronic conduction (such as Ti^3+^ in the LATP system or Li–Ge alloy in the LAGP system) can lead to electron leakage through the interface, triggering persistent side reactions and causing gradual degradation of the solid electrolyte from the near‐interfacial region to even the bulk region of the solid electrolyte. For example, Amine et al. showed that under 80°C cycling, Ge reduction occurred at the Li/LAGP interface to form an amorphous Li‐Ge phase. It was also gradually reduced from the interfacial region to the bulk LAGP, eventually forming a Ge‐rich layer in the center of the LAGP. Kang et al. also demonstrated that when an external current is applied to the Li|LAGP|Li cell, most Li^+^ and electrons meet at the Li/LAGP interface, acting as a “new” negative electrode. The reduction of Li^+^ at the interface leads to the formation of additional Li oxide‐related compounds, resulting in local volume expansion and thus a coupled electro‐chemo‐mechanical failure mode.

Within the SEI region, defects such as oxygen vacancies, cation site disorder, and microstructural inhomogeneity generated during synthesis or sintering provide fast diffusion pathways for active species (e.g., metal cations and/or electrons), thus accelerating these reactions. Furthermore, the formation of heterogeneous and spatially inhomogeneous SEI increases the interfacial impedance, leading to battery cell capacity fading. We will discuss more about the impact of an uneven SEI layer in the next section.

#### 3.2.2 NASICON Bulk Degradation Due to NASICON Preparation and Processing

In addition to the intrinsic SEI, which degrades solid electrolytes chemically and/or electrochemically, the formation of secondary phases during NASICON synthesis also plays a critical role in interfacial stability. These secondary phases are strongly influenced by factors such as the choice of precursors, synthesis methods, and processing temperatures. In the following section, we will use LATP as an example to illustrate how synthesis strategies significantly impact the secondary phase‐related failure mechanism. Generally, synthesis methods of NASICON were fit into two categories: (1). solid phase method (solid‐phase synthesis, Spark plasma sintering (SPS), microwave sintering, and hot pressing, etc.); and (2). liquid phase method (sol–gel method and hydrothermal synthesis method).

**FIGURE 3:**
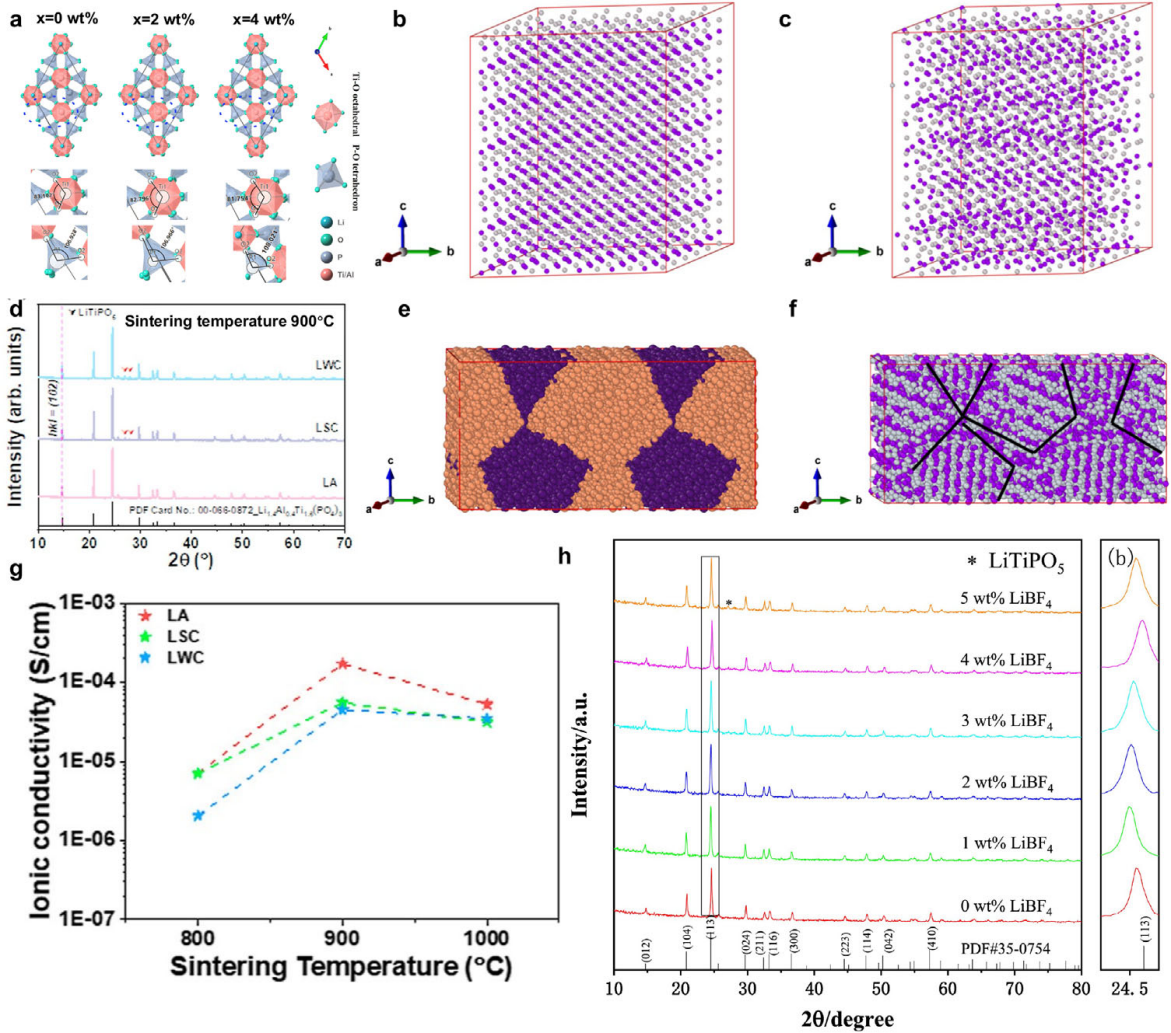
Structural instability and secondary phase formation in doped LATP electrolytes and their implications for ionic transport failure. (a) Change of crystal structure bond Angle of LATP‐*x*SeO_2_ solid electrolyte with different SeO_2_ addition amounts. Reprinted with permission from Yuan et al. [82] Copyright (2025) Elsevier. (b, c, e, f) Molecular dynamics simulated results: initial and final structures for the single‐grain (b, c) and two‐grain polycrystalline (e, f) LATP samples. Here, final structures are obtained after equilibrating the samples with 10 ns MD run. Reprinted with permission [84]. Copyright (2025) American Chemical Society. (d) XRD pattern of LATP_Amorphous (LA), LATP_Semi‐Crystalline (LSC) and LATP_Well‐Crystalline (LWC) sintered at 900°C. Reprinted with permission from Park et al. [87] Copyright (2023) American Chemical Society. (g) Total Li^+^ ion conductivity of all samples by sintering temperature. Reprinted with permission from Park et al. [87] Copyright (2023) American Chemical Society. (h) PDF card (35–0754) and XRD patterns of LATP‐x wt% LiBF_4_ (*x* = 0–5) and magnified XRD patterns for the (113) diffraction peaks. Reprinted with permission from Dai et al. [88]. Copyright (2021) Elsevier.

First, we focus on the impurity formed through solid‐state synthesis. This approach is commonly used to obtain polycrystalline ceramics, in which the raw materials are mixed and then subjected to calcination and sintering processes. Gunamony et al. [81] synthesized LATP using a solid‐phase method and found that after sintering at 800°C–1100°C for 6 h, the impurity of LiTiPO_5_ and TiO_2_ was formed. Yuan et al. [82] found that for Se‐doped LATP, when the doping amount is high (e.g., 4 wt.%), some Se^4+^ ions are converted into Se^6+^ ions and replace P^5+^ ions (Figure 3a). This promotes the precipitation of the second phase LiTiPO_5_ and hinders the migration of Li^+^.

SPS and microwave sintering are both rapid sintering processes that ensure a tight solid‐solid interface between LATP particles. The SPS technique is a pressure‐assisted method in which a direct current is applied to generate heat through the Joule effect. Compared to conventional sintering processes (e.g., isothermal sintering), the SPS technique can significantly reduce sintering time (from hours to minutes) and achieve higher relative density at lower temperatures. However, impurity formation is still an issue here. Courbaron et al. [83] syntheized LATP via SPS and realized that after sintering at 750°C for 5 min, there is still an AlPO_4_ impurity phase, although the secondary phase content is smaller than that under the traditional sintering method. Ghosh et al. fabricated GB‐engineered LATP ceramics using conventional isothermal (CIS) and SPS methods [84]. Rietveld refinement analysis of X‐ray diffraction patterns revealed the absence of any secondary phases in LATP‐SPS (900°C, 5 min, 40 MPa), while very small amounts of LiTiOPO_4_ were observed in the LATP‐CIS (950°C, 10 h, Ar atmosphere) ceramic sample. To understand the difference in Li^+^ conductivity within grains and at grain boundaries, Li migration was investigated computationally (Figure 3b,c,e,f). The slightly higher first peak in the polycrystalline sample indicates localized Li^+^ accumulation at the grain boundaries. While the long‐range order of Li^+^ in the single crystal sample hinders their diffusion, the accumulation of Li^+^ at the grain boundaries and their liquid‐like distribution in the polycrystalline sample enhance ion transport. The computational results agree well with experimental analysis, demonstrating changes in the K‐edge structure observed in both LATP‐CIS and LATP‐SPS samples, indicating the presence of Li‐rich grain boundaries in the latter. Microwave heating is a volumetric heating process that absorbs microwave frequency radiation energy. This volumetric heating enables the sample to be crystallized at a faster heating rate, which can significantly reduce the formation of secondary phases. The sample no longer needs to be heated and cooled slowly to avoid stress caused by thermal gradients. Conventional heating products begin to form a secondary phase of AlPO_4_ at 900°C; in contrast, AlPO_4_ only appears in microwave‐synthesized samples crystallized at 1000°C [85]. Compared with microwave heating, SPS technology is expensive, the equipment is complex, and the sample size is limited. However, SPS can achieve higher density, faster heating rate, and higher controllability.

Second, we will discuss liquid‐phase synthesis methods. The sol–gel method is a typical method for preparing glass‐ceramics; however, unlike the solid‐phase method, it can produce nano‐sized LATP particles at lower temperatures. Liu et al. prepared LATP using a modified citric acid‐assisted sol–gel method [86]. The method involves a two‐step thermal treatment, in which the dry gel is first calcined in argon (500°C) and then in air (700°C–950°C). Above 900°C, the AlPO_4_ impurity is formed, while below 800°C, Li_4_P_2_O_7_ impurities can be seen, with a duration of 2 h for both conditions. Park et al. [87] found that a small amount of LiTiPO_5_ impurity phase (Figure 3d) was present in both semi‐crystalline and well‐crystalline LATP, which may reduce the ionic conductivity (Figure 3g) Dai et al. synthesized LATP using sol–gel method and added LiBF_4_ as a sintering aid [88]. The total ionic conductivity of LATP sintered pellets obtained when LiBF_4_ was added in an amount of 3 wt% reached a maximum of 0.85 mS cm^−1^. However, when LiBF_4_ was added in excess (for example, 5 wt%), a secondary phase LiTiPO_5_ was produced (Figure 3h). resulting in a significant reduction in ionic conductivity of approximately 25% compared with the sample containing 3 wt% LiBF_4_.

In brief, based on the published literature, the secondary phases produced during the synthesis or sintering of LATP are primarily AlPO_4_, LiBiO_2_, TiO_2_, Li_4_P_2_O_7_, and LiTiPO_5_. They can easily lead to interface unevenness and cracks or hinder the migration of metal ions. These secondary phases often introduce interfacial and structural inhomogeneities, thereby increasing interfacial impedance and hindering the uniform transport of Li^+^. The physical properties (ionic and electronic conductivity) of these secondary phases determine their impact on the performance of solid electrolytes. Ionically insulating secondary phases (AlPO_4_, Li_4_P_2_O_7_, and LiTiOPO_4_) block lithium‐ion transport pathways, reducing overall ionic conductivity and increasing interfacial overpotential, thereby accelerating electrochemical instability and dendrite permeation. Conversely, electronically conductive secondary phases (LiTiPO_5_, TiO_2_) promote electron leakage through grain boundaries, inducing localized metal deposition and promoting interfacial redox reactions, ultimately leading to metal dendrite growth and mechanical failure of the solid electrolyte. Therefore, suppressing the formation of secondary phases with unfavorable electronic or ionic properties is crucial to ensuring the electrochemical and mechanical integrity of solid‐state battery systems. However, current studies have also found that some second phases can have good ionic conductivity, which can improve the overall conductivity of solid electrolytes. At the same time, the formation of specific secondary phases can increase the sintering density, enhance interfacial contact, and form a good interface with high dendrite tolerance. We will discuss the positive impact of certain secondary phases in Section 5.

### 3.3 Mechanical Cracking and Dendrite Formation

In addition to chemical and electrochemical degradation at the NASICON–metal interface (e.g., SEI formation, interfacial reduction, and the formation of undesirable secondary phases), interfacial mechanical instability plays an equally critical role. Non‐uniform interphase formation induces electrochemical heterogeneity at the NASICON/metal interface during repeated cycling, which leads to localized current focusing and stress concentration, thereby triggering crack initiation and providing preferential pathways for metal dendrite penetration. These coupled phenomena significantly highlight the importance of viewing interfacial failure not only from chemical and electrochemical perspectives, but also from a mechanics perspective. In this section, we will explore the potential mechanisms of mechanical failure in the NASICON system and their impact on the integrity of solid‐state batteries.

**FIGURE 4:**
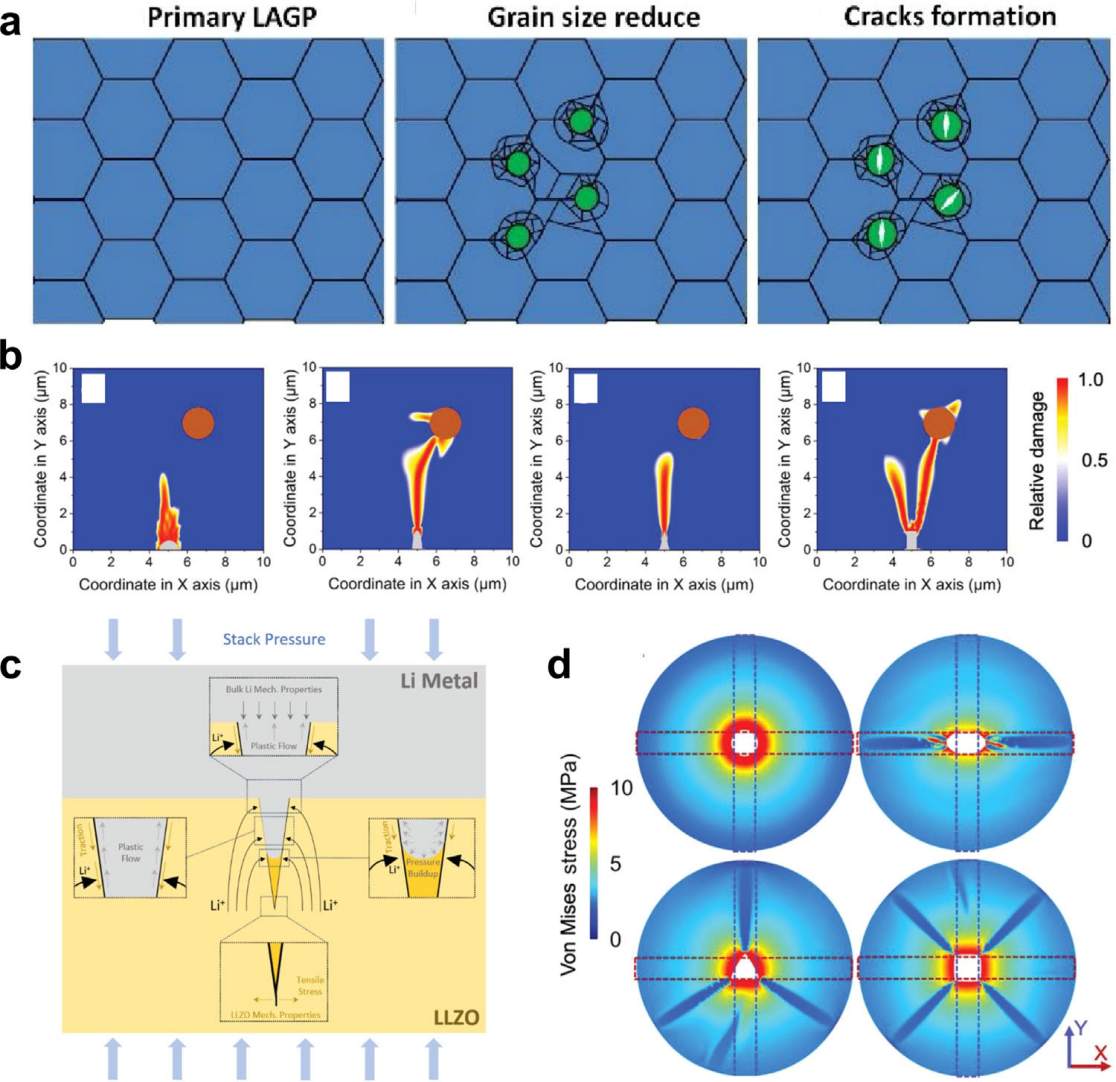
Stress evolution and crack‐driven failure mechanisms in NASICON. (a) Schematic illustration of the NASICON failure process. Reprinted with permission from He et al. [89]. Copyright (2021) American Chemical Society. (b) Visualization of the corresponding damage region with the interfacial defect in the geometry of semi‐sphere, semi‐ellipse, pyramid, and cube. Orange circles represent the internal void. All data are collected at a calculating time of 12 s. Reprinted with permission from Xiong et al. [90]. Copyright (2023) John Wiley and Sons. LLZO wording in the figure refers to Li_6.5_La_3_Zr_1.5_Ta_0.5_O_12_. (c) Schematic showing possible mechanical mechanisms associated with electrochemical plating in a surface crack/flaw of an oxide electrolyte. Due to the geometry of the electrolyte/electrode interface inside the flaw, current redistribution results in additional Li plating inside the surface flaw. The electrochemically‐induced volume expansion that occurs here leads to pressure buildup in the metal. Reprinted with permission from Cho et al. [91]. Copyright (2022) John Wiley and Sons. (d) Visualization of the von Mises stress around the Li filaments with the geometries of cylinder, elliptical cylinder, triangular prism, and cube. Reprinted with permission from Xu et al. [92]. Copyright (2022) John Wiley and Sons.

The formation of the SEI at the NASICON‐metal electrode interface is often spatially heterogeneous due to differences in local composition, surface energy, and defect distribution. This heterogeneity leads to local differences in interfacial chemical reactivity and mechanical properties. In particular, the non‐uniform growth of the SEI introduces regions of differential volume expansion on the NASICON surface. He et al. studied the decomposition products of LAGP solid‐state batteries after charge and discharge [89]. The results show that due to the limited inherent toughness of NASICON, the accumulation of such stresses over time, especially under repeated plating/stripping cycles, may exceed the fracture strength of the ceramic electrolyte, thereby initiating interfacial cracks and even leading to crack propagation throughout the bulk. (Figure 4a) After cycling, LAGP decomposes to form mainly Li_4_P_2_O_7_, Li_3_PO_4_, Ge_5_P_6_O_25_, AlPO_4_, and GeO with the help of Li^+^ NASICON and free electrons from the vacancies and grain boundaries. Finite element method simulations show that the volume expansion caused by the decomposition products (Li_4_P_2_O_7_, Li_3_PO_4_, and AlPO_4_) leads to maximum internal stresses of 2.5–125 GPa at different Li excess ratios ranging from 0 to 6. This far exceeds the failure stress of LAGP (0.788 GPa) and leads to the formation and propagation of cracks in the solid electrolyte during long‐term cycling.

In addition to the SEI, the interfacial defects and internal pores formed during the sintering of the solid electrolyte are also crucial to its failure, due to the limitations of the synthesis and assembly methods. Interface defects were abstracted into typical geometric shapes such as hemispheres, semi‐ellipses, pyramids, and cubes. Xiong et al. established an electrochemical mechanical model and showed that the internal stress of the solid electrolyte caused by the electrodeposition of Li metal at interface defects of different shapes is closely related to the geometric shape of the defect (Figure 4b) [90]. Among them, the cubic defects produce the largest stress inside the solid electrolyte, contributing more than 2 MPa. At the same time, there is a competitive relationship between the defects and the damage of the internal voids. The increase in the number of internal voids will also weaken the mechanical properties of the solid electrolyte, making it more susceptible to cracking and resulting in damage.

Next, we will discuss how metal plating and stripping cause internal stress and damage in NASICON. Studies have shown that these defects act as nucleation points for metal deposition during the electroplating process, leading to increased interfacial heterogeneity (e.g., more interfacial pores and defects). Cho et al. observed the deformation process of Li metal during plating/stripping, which leads to short‐circuiting of a garnet solid electrolyte using a multi‐beam optical stress sensor (MOSS) measurement [91]. The MOSS characterization technique itself will be discussed in detail in Section 4. Here, we mainly focus on the measurement results. When stress accumulates at the tip of the defect (Figure 4c), cracks appear on the electrolyte surface and continue to expand. As Li dendrite grows further, this local stress field is transmitted to the interior of the electrolyte. These mechanical processes, combined with electrochemical reactions, eventually lead to a sudden short circuit. Although the original work is in the garnet solid electrolyte domain, such a mechanism is transferable to NASICON, as both are oxide solid electrolytes. In addition, the growth of metal dendrites will be promoted with the help of interface defects and internal voids in NASICON.

The formed metal dendrite has a concentrated, localized electric field compared to a planar metal surface, further aggravating non‐uniform deposition and promoting penetration. In solid electrolytes (e.g., NASICON), defects promote current crowding at specific locations, thus promoting the nucleation of dendrites. In addition, non‐uniform SEI formation or local secondary phase deposition will further aggravate the inhomogeneity of ion flux and provide preferential paths for dendrite nucleation and growth. Xu et al. showed that the geometric shape of Li dendrites plays a crucial role in the magnitude of local stress around the dendrites and the corresponding size of solid electrolyte cracks (Figure 4d) [92]. For example, cylindrical‐shaped dendrites give the solid electrolyte the smallest cracks. At the same time, the more voids there are inside the electrolyte, the more cracks are caused by metal dendrites.

### 3.4 Computational and Advanced Characterization Insights

Three classes of computational approaches are commonly employed to investigate the interfacial properties of solid electrolytes such as NASICON [39]. (1) Grand‐potential phase diagram calculations are used to determine electrochemical stability windows and decomposition energies, enabling assessment of thermodynamic compatibility between the electrolyte and electrode materials and guiding the selection of protective coating layers [43, 93, 94]. This approach has been briefly discussed in Section 3.1. (2) Nudged Elastic Band (NEB) calculations are applied to quantify ion migration energy barriers along specific diffusion pathways, providing insight into elementary hopping processes and rate‐limiting steps for ion transport across bulk or interfacial regions. (3) Ab initio molecular dynamics (AIMD) simulations are used to capture finite‐temperature ion migration pathways, diffusion mechanisms, and dynamic structural fluctuations, allowing direct evaluation of ion mobility and interfacial transport behavior beyond static energy landscapes. Together, NEB and AIMD calculations enable a comprehensive understanding of ionic conductivity by linking local migration barriers with collective transport dynamics. By combining these computational techniques, NASICON interfaces can be systematically optimized through compositional tuning or interfacial coating design to achieve improved thermodynamic stability and ionic conductivity. Moreover, multiple characterization techniques are often combined with computational approaches to better understand interfacial revolution and degradation mechanisms [95]. For example, techniques such as X‐ray photoelectron spectroscopy (XPS), scanning electron microscopy (SEM)/transmission electron microscopy (TEM), and electrochemical impedance spectroscopy (EIS) are frequently employed to characterize the interphase to reveal its chemical composition, morphology, and interfacial resistance, respectively. Complemented by thermodynamic calculations, the stability between NASICON solid electrolytes and the metal anode, as well as the interphase formation, can be comprehensively elucidated [44]. A detailed discussion of advanced characterization techniques is provided in the next section.

## 4 Experimental Characterization Techniques for Interfacial Behavior

**FIGURE 5:**
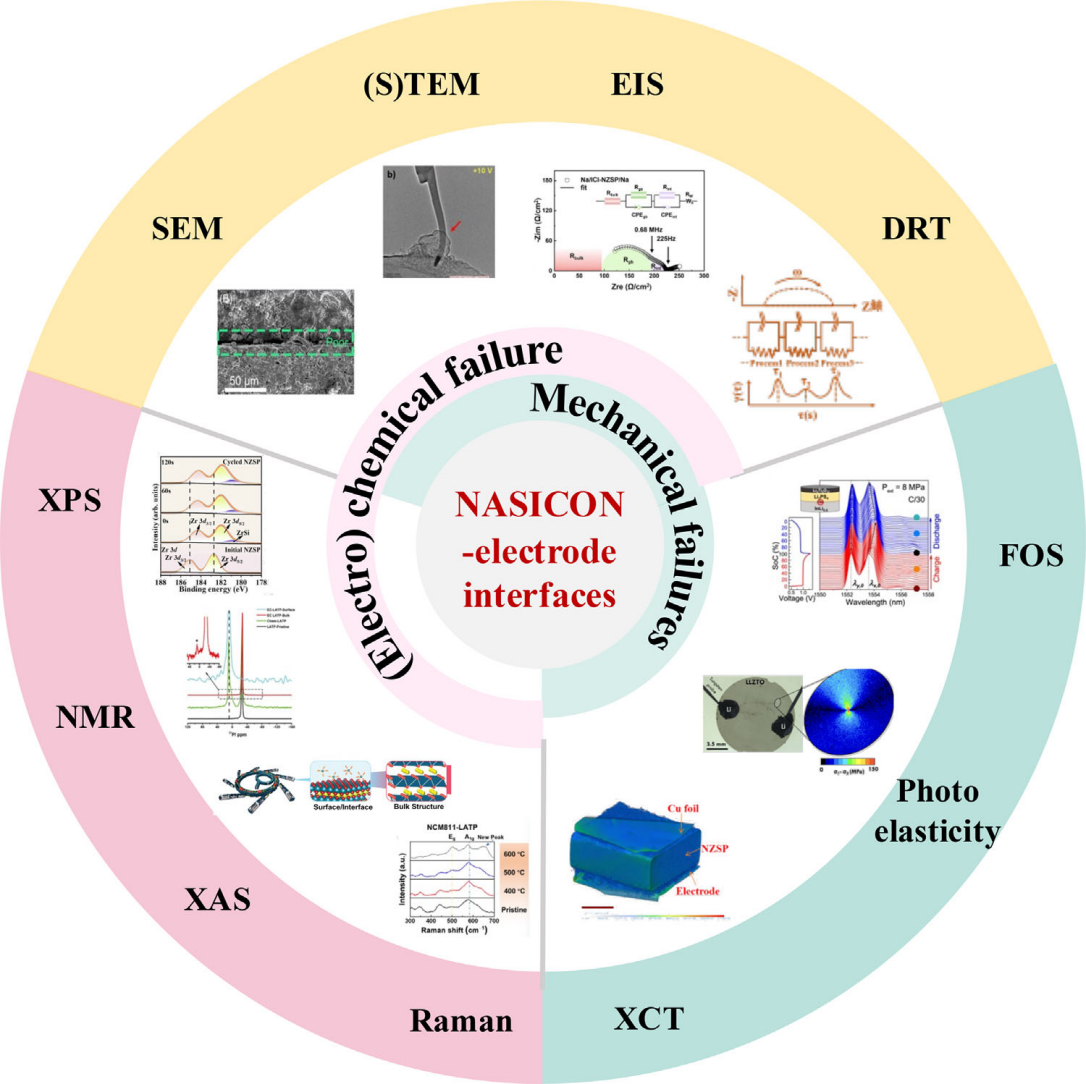
Experimental characterization of interfacial degradation evolution at NASICON/electrode interfaces. Reprinted with permission from Blanquer et al. [96]. Copyright (2022) Springer Nature; reprinted with permission from An et al. [97]. Copyright (2025) Springer Nature; reprinted with permission from Athanasiou et al. [98]. Copyright (2024) Elsevier; reprinted with permission from Li et al. [99]. Copyright (2022) John Wiley and Sons; reprinted with permission from Li et al. [100]. Copyright (2025) Springer Nature; reprinted with permission from Qiu et al. [101]. Copyright (2025) John Wiley and Sons; reprinted with permission from Zhu et al. [102]. Copyright (2020) American Chemical Society; reprinted with permission from Wang et al. [103]. Copyright (2025) John Wiley and Sons; reprinted with permission from Fan et al. [104]. Copyright (2025) John Wiley and Sons; reprinted with permission from Dai et al. [105]. Copyright (2025) John Wiley and Sons.

The complex failure mechanisms at solid electrolyte/metal anode interfaces, as discussed above, necessitate a comprehensive understanding of material behavior, as well as the evolution of interfaces and interphases during cell operation. For electrochemical/chemical failure mechanisms, uncovering the structure and composition of the SEI is essential to elucidate the reaction pathways and to understand the roles of the SEI in dendrite formation, charge transfer, and interphase propagation. This requires a combination of multimodal compositional and structural characterization techniques across multiple length scales (Figure 5) including X‐ray diffraction (XRD), X‐ray photoelectron spectroscopy (XPS), X‐ray absorption spectroscopy (XAS), solid‐state nuclear magnetic resonance (ss‐NMR), Raman spectroscopy, scanning electron microscopy (SEM), (scanning) transmission electron microscopy ((S)TEM), energy‐dispersive X‐ray spectroscopy (EDS) etc. As for mechanical failures, morphological evolution (e.g., contact loss and the formation of cracks/pores) and mechanical monitoring are important for understanding failure mechanisms. The former usually involves microscopy or tomography‐based techniques such as optical microscopy, SEM, TEM, and X‐ray micro‐computed tomography (XCT), while the latter relies on in situ or operando strain/stress measurements. In addition, electrochemical methods such as electrochemical impedance spectroscopy (EIS) and distribution of relaxation times (DRT) analysis are powerful tools for deconvoluting interfacial charge transfer and probing interfacial resistances. They are often combined with other characterization techniques to reveal how chemical, electrochemical, and mechanical behaviors affect interfacial charge transfer and, ultimately, overall cell performance.

In this section, we provide an overview of these key characterization techniques (Figure 5) and discuss how they have been applied to investigate the behaviors of NASICON/electrode interfaces and interfacial degradation mechanisms.

X‐ray techniques are widely used to investigate the SEI of solid electrolytes (e.g., NASICON) because they can probe both surface chemistry and bulk structural changes that occur during SEI formation and evolution. Key X‐ray techniques, including XRD, XPS, XAS, and XCT, collectively enable comprehensive analysis of interfacial phenomena by revealing chemical compositions, electronic structures, and 3D morphologies.

**FIGURE 6:**
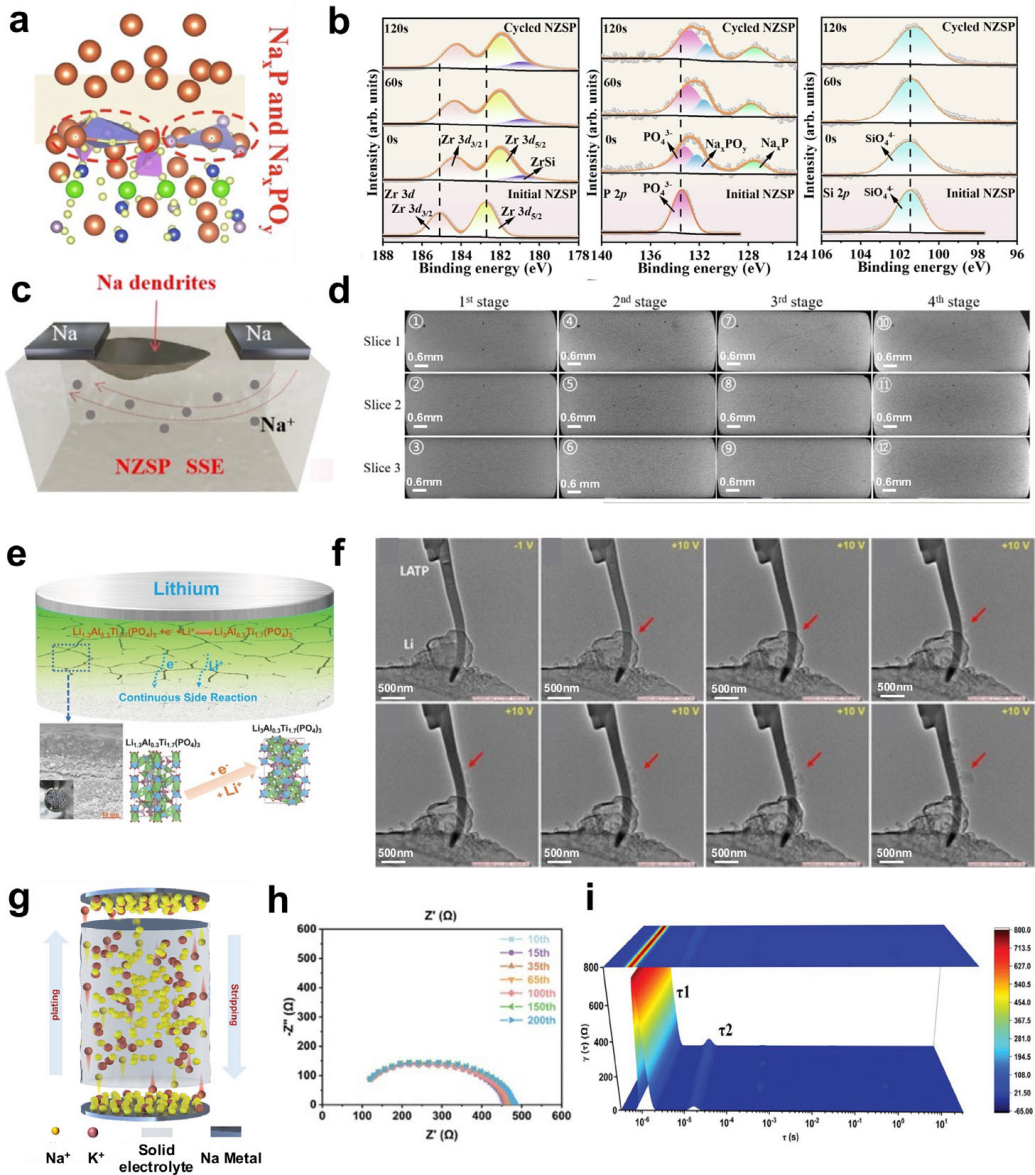
Interfacial degradation and failure mechanisms at metal | solid electrolyte interfaces. (a) Schematic atomic structure of Na|NZSP system at post‐reaction. Reprinted with permission from Li et al. [100] Copyright (2025) Springer Nature. (b) XPS deep etching spectra of Zr 3d, P 2p, and Si 2p before and after Na plating/stripping of NZSP. Reprinted with permission from Li et al. [100] Copyright (2025) Springer Nature. (c) Schematic illustration of Na dendrite penetration through NZSP. Reprinted with permission from Wang et al. [103]. Copyright (2025) John Wiley and Sons. (d) In situ micro‐CT test of the crack‐propagation process in the NZSP block at different current density. Reprinted with permission from Wang et al. [103] Copyright (2025) John Wiley and Sons. (e) Schematic illustration of chemical mechanical failure at Li|LATP interface. Reprinted with permission from Zhu et al. [102] Copyright (2020) American Chemical Society. (f) In situ TEM images of the delithiation process of LATP. Reprinted with permission from Zhu et al. [102] Copyright (2020) American Chemical Society. (g) The schematic diagram of ion migration during the Na plating/stripping process of Na|NZSP‐0.005K|Na. Reprinted with permission from Li et al. [124] Copyright (2025) John Wiley and Sons. (h,i) The EIS and DRT of Na|NZSP‐0.005K|Na at various cycles. Reprinted with permission from Li et al. [124] Copyright (2025) John Wiley and Sons.

XPS, as a highly surface‐sensitive characterization technique with detection limits extending to approximately 10 nm depth, serves as a critical tool for probing chemical states and elemental distributions at NASICON–electrode interfaces. The exceptional sensitivity of this technique to redox‐state shifts as small as 0.1 eV makes it indispensable for identifying interfacial decomposition products that indicate electrochemical and chemical failure mechanisms. At unmodified NASICON/electrode interfaces, XPS analysis is often used to reveal the formation of various degradation products arising from interfacial instability. For example, XPS studies of LATP (Li_1+x_Al_x_Ti_2‐x_ (PO_4_)_3_) provide clear evidence of Ti^4+^ reduction to Ti^3+^ by metallic Li during electrochemical cycling, representing a fundamental chemical failure mechanism that compromises the structural integrity of the interface. Similarly, in LAGP (Li_1+x_Al_x_Ge_2‐x_ (PO_4_)_3_), He et al. observed characteristic peaks of Ge^2+^ after electrochemically cycling for 80 times, suggesting the generation of a reduced specie of GeO. These reduction reactions not only demonstrate chemical instability but also contribute to the formation of electronically conductive phases that can facilitate dendrite propagation. Sodium‐based NASICON systems exhibit analogous interfacial degradation patterns. Li et al. characterized the interfacial reaction products between NZSP and metallic sodium through XPS depth etching analysis, which reveals the shift of Zr 3d, P 2p, and Si 2p peaks to lower binding energies and the formation of decomposition products such as ZrSi, Na_x_PO_y_, and Na_x_P (Figure 6a,b) [100]. This indicates that metallic Na disrupted the PO_4_ tetrahedral structure in NZSP. The resulting formation of electronically conductive ZrSi promotes the propagation of interfacial reactions and ultimately leads to solid‐state battery failure (Figure 6a). The XPS findings across both lithium‐ and sodium‐based NASICON systems demonstrate the essential role of this analytical technique in identifying the chemical composition and temporal evolution of interfacial degradation products, thereby providing critical insights into the factors that directly govern solid‐state battery performance and operational longevity.

XAS, including X‐ray absorption near‐edge structure (XANES) and extended X‐ray absorption fine structure (EXAFS), has emerged as a pivotal analytical technique for investigating NASICON interface phenomena, owing to its element‐specific sensitivity and exceptional capability for probing local structural environments. Recent investigations using XAS have yielded significant breakthroughs in understanding the behavior of the NZSP/Na metal interface. Huang et al. [106] employed in situ micro‐X‐ray absorption near edge structure (µXANES) mapping at the Zr K‐edge to achieve precise chemical environment analysis within NZSP/Na interfacial regions. At the Na/NZSP interface, the Zr K‐edge exhibits a low‐energy displacement of merely 2–3 eV, indicating the formation of a self‐limiting reductive passivation layer composed of Na_2_O, Na_2_O_2_, NaSi, and Na_3_P. Concurrently, the Zr coordination environment within the NZSP bulk remains invariant, demonstrating that the bulk phase undergoes no sustained reduction. Nevertheless, a pronounced metallic Na signal emerges in the interfacial region, directly corresponding to dendrite penetration along grain boundaries. X‐ray absorption spectroscopy, therefore, unambiguously identifies the failure mechanism as rapid dendrite propagation through grain‐boundary channels beneath the preexisting passivation layer rather than decomposition of the NZSP. Moreover, XAS techniques have proven equally valuable for investigating interface failure mechanisms between lithium‐based NASICON electrolytes and cathode materials. Masuda et al. [107] implemented a synergistic analytical approach that combined Ti K‐edge and Co K‐edge spectroscopy to systematically track the chemical evolution processes during high‐temperature sintering of LiCoPO_4_ (LCP) and LATP. The investigation revealed that Ti K‐edge XANES spectra from LATP electrolyte remained invariant throughout high‐temperature processing, whereas Co K‐edge spectra from LCP exhibited pronounced changes at temperatures exceeding 700°C. This arises from the formation of an amorphous reaction layer comprising Li_3_PO_4_ and CoO phases at the LCP/LATP interface upon heating. The comprehensive analysis of NASICON interface behavior through XAS techniques confirms the method's unique ability to distinguish structural differences between bulk materials and interfacial regions while detecting trace reaction products and amorphous phases that remain beyond the detection limits of conventional diffraction techniques. The element‐specific nature of XAS enables the selective monitoring of individual components within complex interfacial systems, providing mechanistic insights that are unattainable through alternative characterization approaches.

XCT employs high‐intensity monochromatic X‐rays to achieve non‐destructive detection and 3D reconstruction of microstructure, offering nanometer‐scale spatial resolution and in‐situ dynamic monitoring capabilities. These unique characteristics enable XCT technology to effectively distinguish multiphase structural interfaces in solid electrolyte systems, providing quantitative analysis of critical parameters such as interfacial contact areas, porosity variations, and crack propagation pathways. For example, Wang et al. [103] employed in‐situ XCT methodology to comprehensively characterize sodium dendrite penetration and crack propagation mechanisms within NZSP (Figure 6c,d). Their investigations revealed a strong correlation between sodium dendrite creep stress and local current density distribution, elucidating a mutually driving coupling mechanism between sodium dendrite penetration and crack propagation. Furthermore, Li et al. [99] utilized synchrotron XCT to conduct 3D microstructural analysis of LATP‐based composite cathodes. The results reveal severe interfacial contact defects between LTAP and the active material Li (Ni_1/3_Mn_1/3_Co_1/3_)O_2_ (NMC). Even under high‐pressure preparation conditions, LTAP achieved coverage of only 59% of the NMC particle surface area. Concurrently, XCT measurements showed that the ionic transport tortuosity of the LTAP phase exceeded theoretical values by 2–4 times, resulting in significant obstruction of Li^+^ ion transport pathways. These findings point out poor inter‐particle contact as the key factor that causes compromised capacity, poor rate capability, and shortened cycle life of LATP‐based solid‐state batteries. These examples demonstrate the unique technical value and application significance of XCT technology in the degradation mechanism study of NASICON‐based solid electrolytes.

Beyond X‐ray techniques, advanced electron microscopy (e.g., SEM, (S)TEM, EDS) is essential for characterizing the morphology, structure, and composition of solid electrolyte‐electrode interfaces, spanning scales from atomic to micro.

SEM generates high‐resolution morphological information by scanning sample surfaces with focused electron beams, enabling direct observation of interfacial contact states and morphological variations. This technique provides crucial microscopic evidence for evaluating the quality of interfacial contact. (S)TEM features atomic‐level resolution by transmitting electron beams through specimens, enabling in‐depth analysis of interfacial layer microstructural evolution, phase transformation processes, and crystallographic characteristics, thereby providing direct microscopic evidence for elucidating fundamental interface failure mechanisms. EDS functions as a compositional analysis technique that determines elemental composition and distribution by detecting characteristic X‐rays, operating in conjunction with SEM and TEM to provide comprehensive analytical capabilities. These complementary techniques provide comprehensive structural and compositional information, enabling a thorough understanding of the microscopic mechanisms underlying NASICON/electrolyte interface failure. This establishes them as essential characterization tools for developing high‐performance NASICON‐based solid‐state batteries. For the NASICON/anode interface, Li et al. [108] employed cross‐sectional SEM‐EDS analysis to confirm that ultrasonically welded Na/Au‐NZSP interfaces maintained intimate contact states after 900 cycles. Similarly, Lei et al. [109] employed cross‐sectional SEM analysis to compare the interface changes in *p*‐type semiconductor dipotassium rhodizonate (K2C6O6) (DKR)‐coated LATP versus pristine LATP electrolytes before and after cycling, revealing a stable interface enabled by the DKR buffer layer. Furthermore, Ortmann et al. [110] employed SEM and high‐angle annular dark‐field (HAADF)‐STEM techniques for in‐depth analysis of NZSP anodic interfaces, observing the formation of signal‐enhanced thin layer structures at the interfaces while maintaining excellent structural continuity between the interfacial layers and the NZSP. Subsequent EDS compositional analysis revealed that these interfacial layers primarily consisted of sodium and oxygen elements, with undetectable amounts of zirconium and silicon, indicating that NZSP underwent minimal chemical decomposition. Moreover, in‐situ TEM has been applied to investigate the impacts of high electronic conductivity of the interphase between LATP and Li metal on the deposition of the Li [102], as shown in Figure 6e,f. We note, however, that despite its power, the application of electron microscopy to NASICON systems has been largely limited by their inherent electron beam sensitivity. Careful sample preparation and the use of low‐dose techniques are essential to characterize the materials and interfaces in a near‐native state [111].

ss‐NMR and Raman spectroscopy techniques serve as important structural characterization methods, providing unique atomic/molecular‐level insights for revealing the interface failure mechanisms of NASICON. This capability stems from their ability to directly detect local structural changes, ionic migration behaviors, and interfacial chemical reaction processes of materials at the atomic/molecular scale. Specifically, ss‐NMR spectroscopy exploits nuclear resonance phenomena in magnetic fields to precisely identify phase transitions and interfacial reaction products in NASICON materials through the detection of chemical shifts, relaxation times, and peak morphology changes of specific nuclei. Complementarily, Raman spectroscopy sensitively detects structural phase transitions, chemical bond variations, and interfacial chemical reactions in NASICON systems by analyzing changes in molecular vibrational modes. For example, Ren et al. [112] investigated the local structure and Na^+^ dynamics of NaF‐doped NZSP (NZSP‐NaF) through a comprehensive solid‐state NMR (ss‐NMR) study combining ^31^P, ^29^Si, and ^23^Na. Complementary Raman spectroscopic analysis of NZSP‐NaF revealed the emergence of a new P‐O‐P bending vibrational peak at 105.17 cm^−1^. These observations collectively confirmed that NaF incorporation triggered the structural transformation of NZSP from an ordered crystalline to a locally disordered amorphous configuration, thereby facilitating Na^+^ ion transport and promoting uniform ionic distribution at interfaces. Moreover, the ss‐NMR and Raman spectroscopy techniques have proven instrumental in identifying interfacial reaction products in NASICON systems. Zhu et al. [102] employed ^7^Li, ^27^Al, and ^31^P ss‐NMR to investigate LATP/Li interfaces and discovered the formation of lithiated phases Li_3_Al_0.3_Ti_1.7_ (PO_4_)_3_. This interfacial transformation resulted in an enhancement of interfacial electronic conductivity by at least three orders of magnitude, subsequently triggering continuous side reactions and degradation of electrochemical performance. In situ TEM observations further validated the conclusions derived from NMR analysis, directly visualizing lithium dendrite growth from within the electrolyte interior due to interfacial reactions. Dai et al. [105] conducted Raman spectroscopic analysis of composite cathodes comprising LATP and LiNi_x_Co_y_Mn_1‐x‐y_O_2_ (NCM) during co‐sintering processes and observed NCM transformation from layered to spinel phase. Similarly, An et al. [97] employed tip‐enhanced Raman spectroscopy to analyze the interfaces between NZSP and propylene carbonate (PC)‐based liquid electrolytes and discovered that the characteristic Raman peaks red‐shifted from 840 to 830 cm^−1^ with increasing current densities. This spectral evolution indicated strengthened chemical coordination reactions between PC molecules and surface Na^+^ ions on NZSP, leading to Na vacancy formation and subsequent organic molecule decomposition.

**FIGURE 7:**
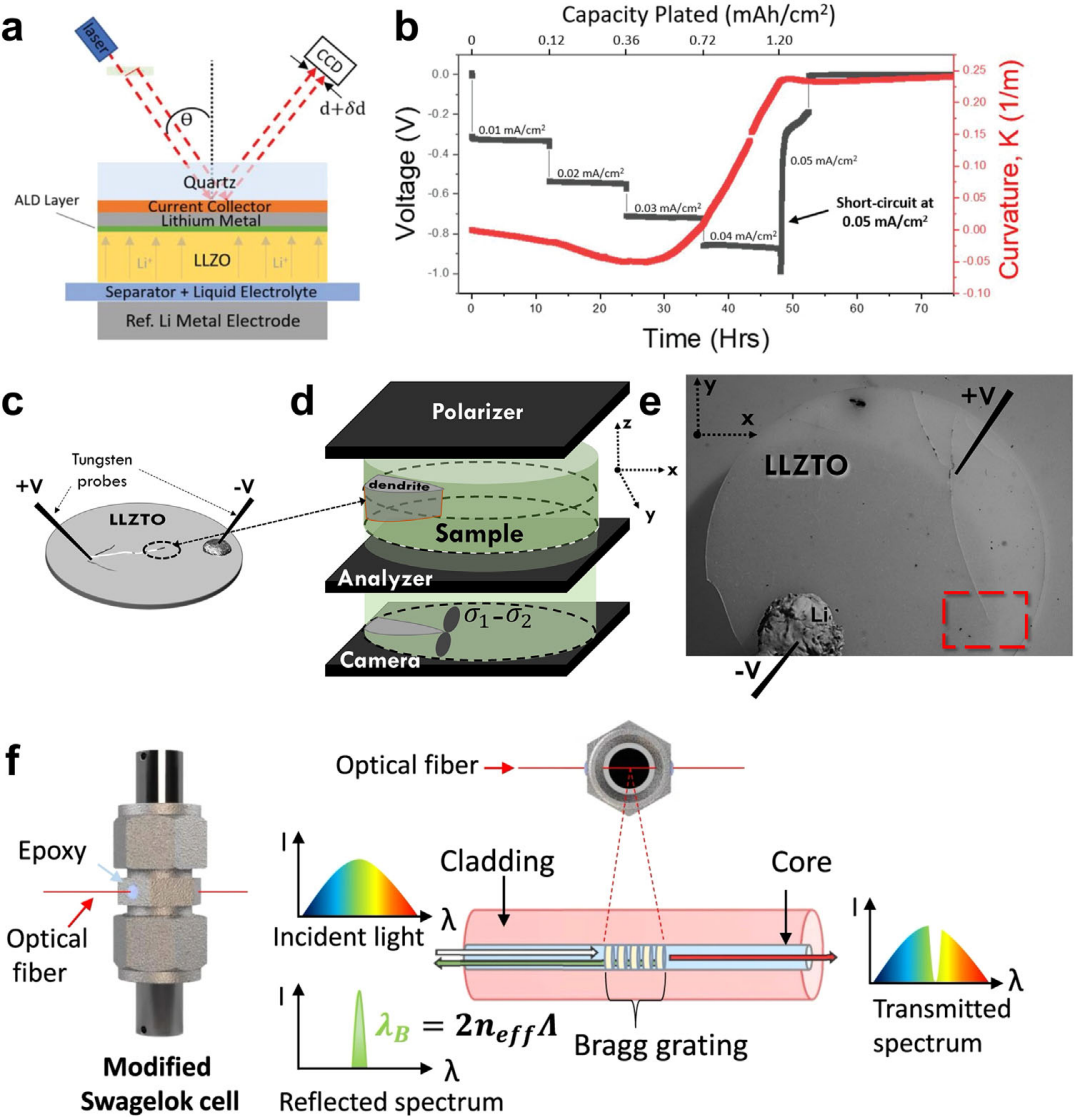
Operando optical and mechanical analysis techniques for monitoring stress evolution and dendrite growth in solid‐state batteries. (a) Schematic showing how the curvature evolution is measured during plating. During the measurement, a set of parallel laser beams is reflected off the reflecting current collector, which tracks the evolving curvature associated with lithium metal during plating. Reprinted with permission from Cho et al. [91] Copyright (2022) John Wiley and Sons. (b) Voltage & curvature versus time & capacity plated of the LLZO/quartz electrode. Black and red lines indicate voltage and curvature profile, respectively. Plating current is applied as a stepwise increase of 0.01 mA cm^−2^ increments every 12 h, starting at 0.01 mA cm^−2^. Reprinted with permission from Cho et al. [91] Copyright (2022) John Wiley and Sons. (c–e) Schematic of the experimental setup and the photoelastic principle allowing for measuring the stress field around a metal dendrite—where the stress represents the difference in in‐plane maximum principal stresses (σ_1_ − σ_2_). Metal electrodes adhered to the surface of a thin LLZTO electrolyte and are polarized to produce in‐plane metal growth. Figures c–e are reproduced from the same reference, and reprinted with permission from Athanasiou et al. [98] Copyright (2024) Elsevier. (c) LLZTO electrolyte experimental testing configuration. (d) A birefringence microscope measuring the corresponding operando stress field (see inset) during dendrite growth. (e) Post‐mortem optical micrograph of a plan‐view cell. (f) Scheme of the integration of an FBG into an in‐house modified Swagelok cell together with the working principle of an FBG optical sensor. Reprinted with permission from Blanquer et al. [96] Copyright (2022) Springer Nature.

To visualize the stress field generated in solid electrolytes by metal dendrite formation and elucidate micro‐mechanisms related to stress development, techniques capable of in‐situ direct stress measurement and observation are essential [113]. A successful in‐situ MOSS setup has been developed that can directly measure curvature changes correlating to stress in real‐time, in conjunction with a homemade electrochemical cell setup [91, 114, 115]. The MOSS technology is particularly valuable because it offers a high degree of sensitivity and precision in detecting even second changes in curvature, which are indicative of stress variations within the material (Figure 7a). This system, combined with a homemade electrochemical cell is adept at monitoring stress evolution and changes during electrochemical cycling. It has proven effective across multiple electrochemical systems, including both electrode and electrolyte materials [91, 116]. During metal plating cycles, an increasing trend in positive curvature value was observed, indicating that the side of the solid electrolyte where lithium was being plated was under tension. Notably, when the solid electrolyte experienced an electrical short due to lithium dendrite growth, the curvature ceased to change and stabilized (Figure 7b) [91]. The stabilization of curvature at the point of electrical shorting suggests a direct link between the mechanical stress in the solid electrolyte and the structural changes caused by metal dendrite penetration. This provided clear evidence that the metal dendrite growth caused the tensile stress generated inside the solid electrolyte. Furthermore, 2D digital image correlation method shows promise in characterizing the spatial distribution of strain at the solid electrolyte/metal‐metal interface [117, 118]. But the lack of standardization in algorithmic approaches can lead to discrepancies in the data interpretation and the accuracy of the strain measurements. While this technique is useful for visualizing strain patterns, careful consideration and calibration are necessary for reliable quantitative analysis.

Operando approaches using external pressure sensors and built‐in fiber optic sensors (FOS) can also track the evolution of stress and strain at the solid electrolyte /electrode interface [96, 118, 119, 120]. For external pressure sensors, it has been successfully applied to SSBs to monitor stress changes due to charge transfer electrochemical reactions and Li dendrite growth in Li metal‐based anodes [118, 120]. For the built‐in FOS, it provides a direct measurement of strain changes around its localized regions without causing chemical or electrochemical reactions with the cell components (Figure 7c–e) [96, 119]. However, implementing FOS involves accommodating their shape at the solid electrolyte /electrode interface, which can result in varying localized stress distributions. The regions near the FOS may experience different stress patterns compared to areas further away from the FOS, potentially affecting the uniformity of stress measurement across the solid electrolyte /electrode interface. This disparity in stress distribution resulting from the physical presence and integration of the FOS needs to be carefully considered when interpreting the data obtained from these sensors.

Alternatively, an operando photoelasticity‐driven approach to visualize the dendrite‐induced stresses built up in solid electrolytes may minimize the side effects on the sensor‐based approaches [98]. Photoelasticity is a whole‐field technique for measuring and visualizing stresses and strains in structures. It is one of the oldest methods for experimental stress analysis, first observed by David Brewster in the early 19th century. This method is particularly effective when applied to transparent crystal materials and amorphous matrices [121]. From the displacement data directly measured by photoelasticity, strain and stress fields can be predicted through analysis by Dally et al. [122]. This methodology can be applied to solid electrolytes to observe highly concentrated strain and stress around lithium dendrites’ tips during the cycling of solid‐state batteries. Using this method, operando measurements of dendrite‐induced stresses have been developed to observe stress concentrations at through‐thickness dendrite tips in oxide solid electrolyte [98]. This approach provides valuable insights into the stress distribution and mechanical behavior of solid electrolytes under real operating conditions, enhancing our understanding of lithium dendrite propagation and its impact on battery performance. A specially designed experimental setup was introduced to easily and conveniently visualize the stress buildup during metal plating (Figure 7f) [98]. While the current version of the photoelasticity‐driven approach for measuring stress build‐up within solid electrolytes necessitates the use of a translucent solid electrolyte for transmission‐based experiments, it offers distinct advantages in allowing for the direct measurement of stress accumulation by mapping stress distribution at the Li dendrite tip region across the solid electrolytes. The value of this technique lies in its ability to provide a clear and localized view of stress dynamics within the solid electrolyte during Li plating.

In addition, electrochemical analysis methods, such as EIS and DRT, are powerful techniques for deconvoluting and quantifying interfacial resistance buildup and monitoring interfacial degradation over time. EIS constitutes a technique that characterizes electrochemical systems by applying a small‐amplitude sinusoidal voltage and measuring the impedance response of the system at different frequencies. This technique provides important information regarding electrode process kinetics, interfacial properties, and transport phenomena. Moreover, the DRT technique serves as an advanced data processing method that converts complex EIS spectra into relaxation time distributions, thus effectively identifying and deconvoluting different electrochemical processes. When applied to NASICON interface analysis, the combined EIS‐DRT approach precisely identifies and quantifies various electrochemical processes occurring at interfaces, including grain boundary responses, interfacial ion transport, charge transfer reactions, and diffusion processes. For example, EIS‐DRT has been applied to compare full‐cell performance between NZSP and NVP cathodes assembled with different interfacial phase modifications [101], revealing the critical role of eliminating electronic conductive components in enhancing the interfacial stability of NASICON. Furthermore, Hsiang et al. [123] conducted comprehensive EIS‐DRT analysis on composite cathodes comprising LATP and LiTi_2_ (PO_4_)_3_ (LTP) processed at various sintering temperatures (700°C, 800°C, 900°C, 1000°C). The analytical results emphasize the need for low‐temperature sintering processes to minimize high interfacial impedance resulting from interfacial reaction byproducts. The application of EIS‐DRT analysis to anode interfaces has yielded equally valuable insights. Li et al. [124] analyzed Na/NZSP/Na and Na/NZSP‐0.005K/Na symmetric cells, with DRT‐EIS analysis successfully identifying two characteristic time constants corresponding to grain boundary responses and interfacial reaction processes (Figure 6g–i). Similarly, Su et al. [31] implemented combined EIS‐DRT analytical techniques to comparatively evaluate interfacial performance between Li|LATP|Li and Li|Ga (NO_3_)_3_@LATP|Li symmetric cells. The comparative analysis demonstrated that Li|Ga (NO_3_)_3_@LATP|Li exhibited fewer DRT peaks associated with interfacial ion transport and reduced peak areas compared to unmodified Li|LATP|Li, suggesting the suppression of side reactions between LATP and metallic Na anodes by Ga (NO_3_)_3_ layers.

Each category of characterization techniques discussed above provides unique insights into the behavior and failure mechanisms of NASICON/electrode interfaces. Achieving a comprehensive understanding, therefore requires integrating multiple analytical techniques. This, in turn, necessitates the development of integrated, multimodal platforms that can correlate data from these different techniques across identical sample regions. Furthermore, advances in in situ or operando approaches are essential for uncovering degradation pathways during cell cycling and elucidating the dynamic interplay between structural, compositional, and electrochemical processes. Moreover, the development of advanced electron microscopy or synchrotron‐based characterization techniques tailored for solid‐state battery research can further enhance our observational depth and resolution, thereby broadening our understanding of buried solid‐solid interfaces.

## 5 Coupled Electro‐Chemo‐Mechanical Interfacial Engineering Strategies

In the past chapters, we have discussed the electro‐chemo‐mechanical impacts on the interface between NASICON‐type electrolytes and batteries. For example, Ti^4+^ in LATP is reduced to Ti^3+^ and Zr^4+^ in NZSP is reduced to Zr^3+^ by simple physical contact with Li and Na metal, respectively. Coupled with the repeated Li deposition/stripping process during battery cell operation, the reaction between Li/LATP will continuously destroy the interface, resulting in a continuous increase in interfacial impedance. The interfacial byproducts also exhibit non‐negligible electronic conductivity, which promotes the nucleation and growth of Li dendrites, ultimately leading to the failure of LATP. Additionally, the inherent mechanical brittleness of LATP renders it susceptible to stress concentration and crack formation during cycling, due to the volume expansion of interfacial reaction products. As the cycle progresses, the continuous evolution of cracks eventually leads to electrolyte fragmentation and mechanical failure.

To realize the practical application of NASICON‐type electrolytes, extensive research has been conducted to improve the interfacial stability between NASICON‐type electrolytes and electrodes. It can be divided into three aspects: (1) Modification of the NASICON solid electrolyte, including element doping and controlling the generation of favorable secondary phases during electrolyte synthesis. (2). Building a 3D solid electrolyte architecture. (3) Composite metal electrode strategies. (4) Design of the interface between NASICON solid electrolytes and electrodes through a composite intermediate layer. (e.g., inorganic surface modification and polymer surface modification)

### 5.1 Intrinsic NASICON Solid Electrolyte Designs

In this section, we focus on how to engineer the Li‐/Na‐based NASICON itself to solve the interface problem between the electrolyte and the metal electrode. It includes chemical‐driven and structure‐driven designs. Chemically, we will discuss how to use doping strategies to mitigate the dendrite issue. The doping strategies are beneficial in the following ways: (i) widening ion migration pathways, leading to a lower overpotential and thus a lower probability of metal dendrite formation; (ii) forming beneficial secondary phases, enhancing grain boundary conductivity and suppressing abnormal grain growth; (iii) promoting metal wetting ability, and suppressing dendrite formation. Considering the benefits of a certain secondary phase, we will also cover the design of an artificial secondary phase to mitigate dendrite issues. Structurally, we will discuss manufacturing 3D structures to mitigate the dendrite issue.

#### 5.1.1 Lithium NASICON Conductor

Ti^4+^ ions in LATP are considered unstable when in contact with Li metal. Similarly, Ge^4+^ in LAGP is susceptible to reduction by Li, degrading interfacial stability. Therefore, strategies focusing on reducing the adverse effects of these transition‐metal elements are highly desirable for mitigating dendrite‐related issues. One effective approach is elemental doping to substitute these transition metals partially. Such doping not only minimizes their detrimental impact on interfacial stability but can also significantly enhance the ionic conductivity of the solid electrolyte. Improved conductivity lowers the interfacial overpotential during cycling, thereby further suppressing dendrite nucleation and growth.

**FIGURE 8:**
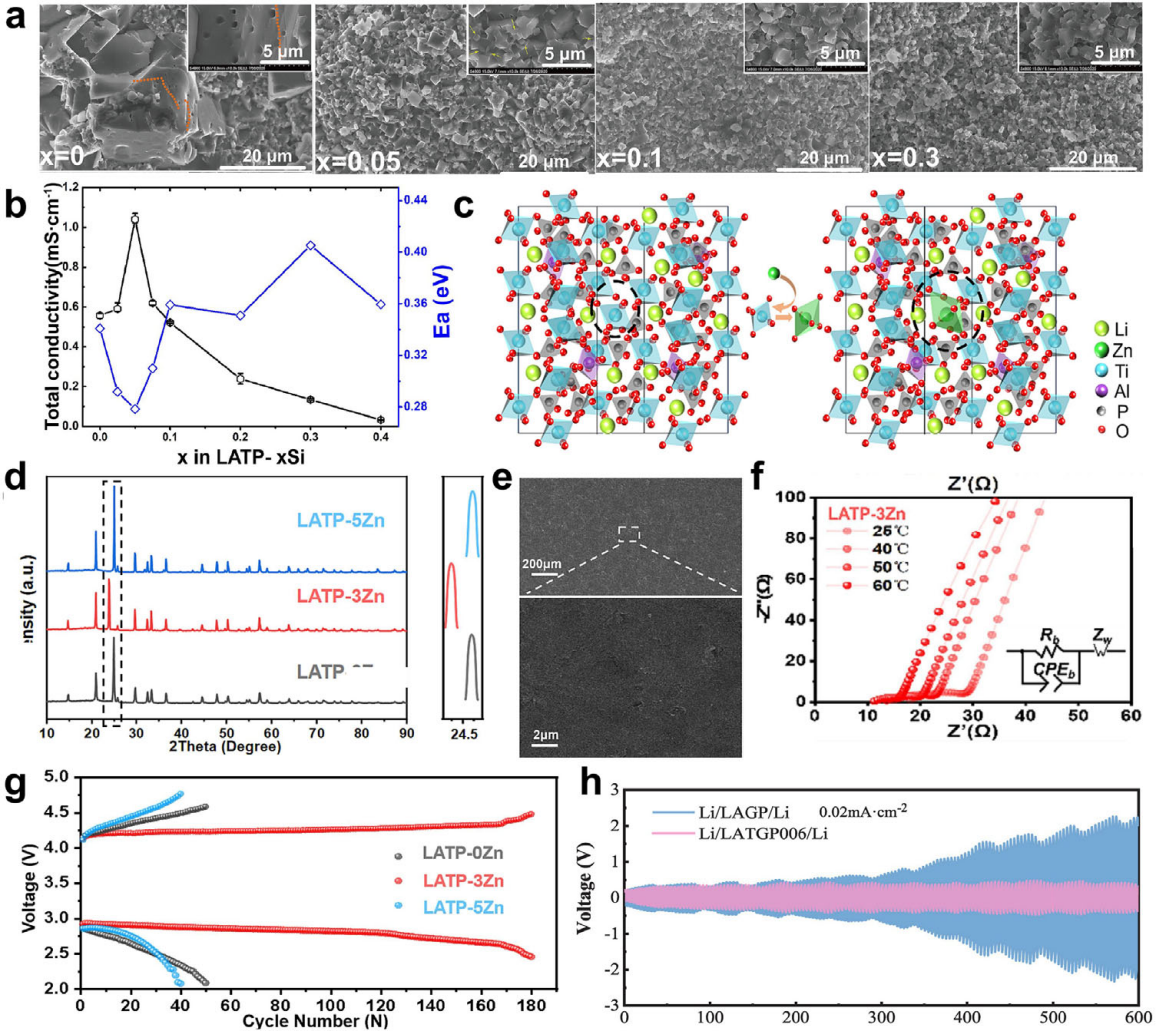
Elemental doping strategies for enhancing Li^+^ NASICON conductors. (a) Cross‐section SEM of LATP‐xSi electrolyte pellet. Inset is the corresponding cross‐section SEM at higher magnification. Reprinted with permission from Zhu et al. [125] Copyright (2022) Elsevier. (b) The variation of total conductivity and activation energy with the increase of silicon doping content. Reprinted with permission from Zhu et al. [125] Copyright (2022) Elsevier. (c) Schematic process for the preparation of the LATP‐xZn solid‐state ceramic electrolyte. Reprinted with permission from Zhu et al. [125] Copyright (2022) Elsevier. (d) XRD patterns of LATP‐xZn solid‐state ceramic electrolyte after discharge and amplified diffraction patterns of solid (113) surface [127]. (e) SEM images of LATP‐3 Zn [127]. (f) EIS of LATP‐3 Zn [127]. (g) the cycling performance of the Li–CO_2_ batteries. Figures d–g are reproduced from the same reference and reprinted with permission from Zhu et al. [127] Copyright (2024) Elsevier. (h) Voltage profiles of the Li|LAGP|Li cell and Li|LATGP006|Li cell at a current density of 0.02 mA cm^−2^. Reprinted with permission from et al. [130] Copyright (2023) Elsevier.

Zhu et al. synthesized a series of Si‐doped LATP samples, Li_1.3+x_Al_0.3_Ti_1.7_Si_x_P_3‐x_O_12_ (LATP‐xSi, *x* = 0–0.4) (Figure 8a) [125]. Unlike NZSP, where Si is doped into tetrahedral P^5+^ sites, causing the unit cell volume to expand. Si doping into LATP displaces Ti^4+^ sites in octahedral sites, causing the unit cell volume to shrink. At low Si doping levels (e.g., *x* < 0.1), a small amount of generated secondary phase of LiTiOPO_4_ suppresses abnormal grain growth and reduces porosity (Figure 8b). In parallel, LiTiOPO_4_ itself is Li‐ion conductive, which contributes to grain boundary conductivity. At *x* = 0.05, LATP‐0.05Si achieves the highest conductivity of 10^−3^ S⋅cm^−1^ at room temperature, which is more than doubled compared with the undoped LATP. In another study, Hupfer et al. artificially introduced LiTiOPO_4_ into LATP and successfully cross‐checked the functionality of the LiTiOPO_4_ secondary phase [126].

An additional example has been provided by Zhu et al., who prepared Zn‐doped LATP, designated as LATP‐xZn, with x ranging from 0 to 5 wt%, via a high‐temperature solid‐phase reaction (Figure 8c) [127]. Their XRD results showed that when the Zn doping level was 3 wt%, the deflection angle was maximum near 24.5°, indicating that Zn^2+^ ions replaced Ti^4+^ ions, resulting in a decrease in the lattice gap (Figure 8d). This contributes to improved ionic conductivity. The authors also demonstrated that LATP‐3 Zn exhibited significantly improved smoothness and flatness of the sample surface, compared to LATP‐0 Zn and LATP‐5 Zn (Figure 8e). The denser surface facilitates uniform Li deposition between the electrolyte and the Li metal, reducing dendrite penetration. It also enhances contact with the Li metal and reduces interfacial impedance. The ionic conductivity of LATP‐3 Zn was 2.45 × 10^−3^ S cm^−1^ at 25°C (Figure 8f). In Li‐CO_2_ battery cell configurations, the LATP‐3 Zn underwent 180 stable cycles without a shorting behavior at a current density of 100 mA g^−1^, a cutoff capacity of 500 mAh g^−1^, and a voltage range of 2.5–4.5 V (Figure 8g).

Similar to LATP, the Al^3+^ and Ge^4+^ in LAGP can be partially replaced by cations with larger ionic radii, thus expanding the Li‐ion migration channels in the structure. Numerous studies have demonstrated that doping with divalent cations can enhance the Li‐ion concentration and widen the diffusion channels, thereby improving Li‐ion conductivity. Si^4+^ can also dope LAGP, but unlike LATP, in LAGP, Si^4+^ replaces the tetrahedron where P^5+^ resides. Pershina et al. prepared Li_1.5+х_Al_0.5_Ge_1.5_Si_x_P_3‐x_O_12_ (0 ≤х ≤ 0.5) by glass crystallization [128]. The Li_1.52_Al_0.5_Ge_1.5_Si_0.02_P_2.98_O_12_ composition crystallized at 750°C exhibited the highest Li^+^ conductivity, at 4.55 × 10^−4^ S cm^−1^ at 25°C.

Nikodimos et al. introduced Mg into LAGP to form Li_1.6_Al_0.4_Mg_0.1_Ge_1.5_ (PO_4_)_3_ (LAMGP) [129]. Due to the large ionic radius of Mg, doping LAGP with Mg increases the Li^+^ concentration, expands lattice parameters, and improves ionic conductivity. Furthermore, the densification caused by doping reduces grain boundary resistance and improves the interface between LAGP and the Li metal. Both effects are beneficial in lowering overpotential and thus metal dendrite formation. Experimentally, the LAMGP electrolyte yields an ultrahigh volumetric ionic conductivity of ∼7.4 mS cm^−1^ (compared to ∼2.9 mS cm^−1^ in undoped LAGP). To further enhance interfacial contact, 5 µL of carbonate‐based liquid electrolyte was added. The Li|LE|LAMGP|LE|Li symmetric cell remained stable for ∼1000 h of cycling at 25°C and a current density of 0.2 mA cm^−2^, with a cumulative total of 200 mA h cm^−2^. Liu et al. used the sol–gel method to dope Te into LAGP and obtained a series of LATGP (*x* = 0, 0.02, 0.04, 0.06, 0.08) materials [130]. XRD results demonstrate that when the doping concentration exceeds 4%, higher doping concentrations do not further increase the lattice volume. This suggests that further increasing the doping concentration will not effectively improve the material's ion transport properties, as additional Te doping introduces new impurity phases. The LATGP006 sample achieved a maximum ionic conductivity of 6.33 × 10^−4^ S cm^−1^. Symmetrical Li|LAGP|Li and Li|LATGP006|Li cells were subjected to galvanostatic cycling. At a current density of 0.02 mA cm^−2^, the polarization voltage of the symmetric Li|LATGP006|Li cell remained within ± 0.5 V after 600 h of cycling (Figure 8h).

**FIGURE 9:**
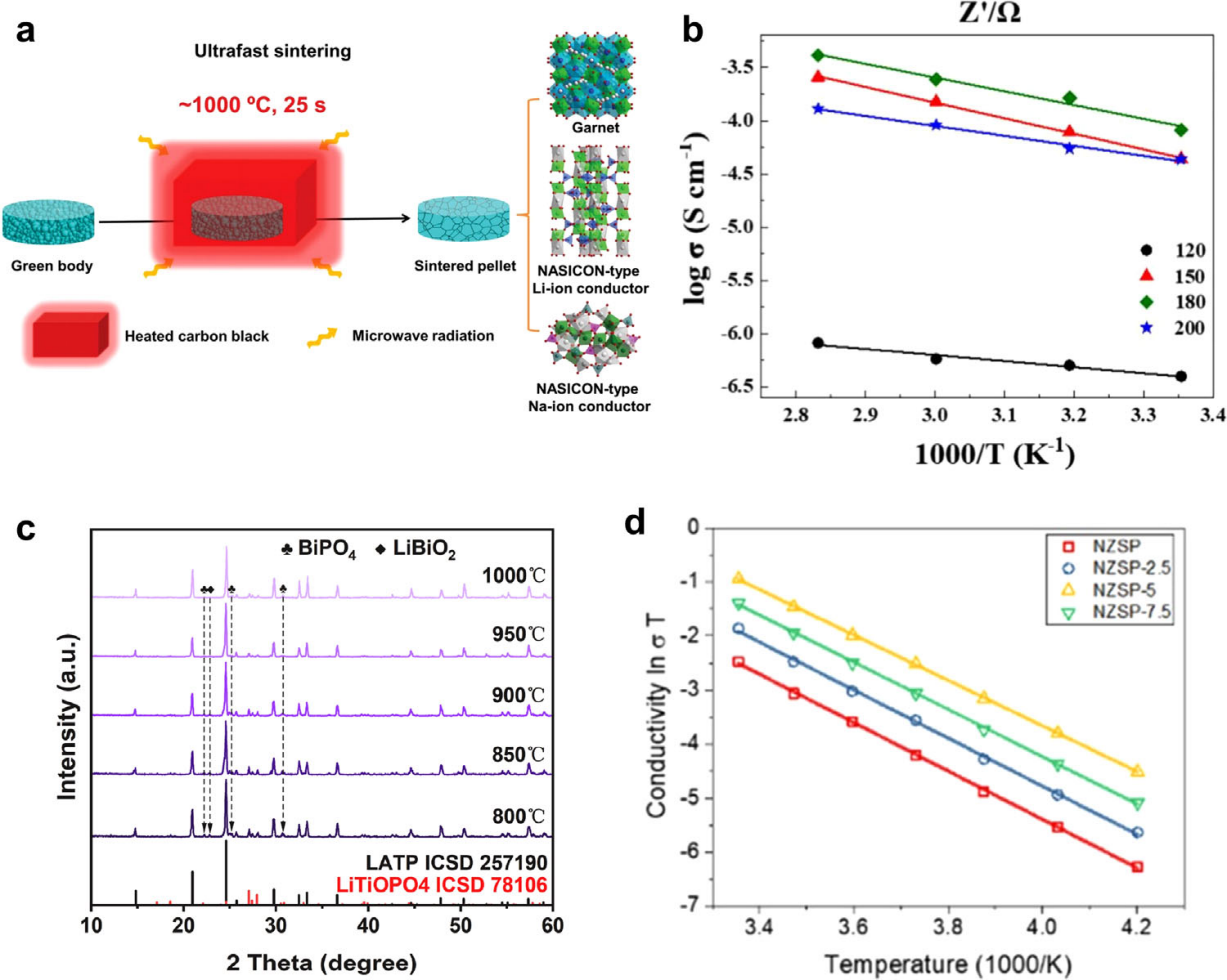
Sintering process engineering of NASICON‐type solid electrolytes and its impact on ionic conductivity. (a) Schematic of the ultrafast sintering and co‐sintering process. Through microwave‐induced carbothermal shock, various ceramic electrolytes and electrode–electrolyte multilayers are ultrafast sintered in ≈25 s. The volatilization of Li/Na and the interdiffusion in electrode/electrolyte interface are effectively suppressed. Reprinted with permission from Chen et al. [131] Copyright (2022) John Wiley and Sons. (b) Ionic conductivity of samples as a function of temperature in the range of 25°C–80°C. Reprinted with permission from Yu et al. [132] Copyright (2024) Elsevier. (c) LATP‐0LB and LATP‐2LB ceramic pellets sintered at different temperatures. Reprinted with permission from Luo et al. [133] Copyright (2022) Elsevier. (d) Total ionic conductivity of electrolyte at different temperatures. Reprinted with permission from Oh et al. [51] Copyright (2019) American Chemical Society.

While compositional modifications such as doping have been widely used to enhance the ionic conductivity of NASICON electrolytes, the role of synthesis strategies is equally, if not more, important. Tailoring synthesis routes allows for better control over crystallinity, phase composition, particle size, and microstructural uniformity, which collectively determine the lithium‐ion transport performance. Chen et al. developed a microwave‐assisted ultrafast sintering technique (MAUST) for sintering various ceramic electrolytes in air using a household microwave oven (Figure 9a) [131]. The relative density of LATP particles obtained by this technique can reach 98.3% ± 3.4%. Compared with conventional sintering (particle density of 96.7% ± 2.8%), MAUST exhibits a more pronounced densification effect. MAUST can simultaneously densify the integration of the electrolyte layer and the electrode layer within 25 s, resulting in a dense interface. They also applied the same technology to the synthesis process of garnet solid electrolyte. The results showed that the garnet solid electrolyte was partially converted into the impurity phase La_2_Zr_2_O_7_ during traditional sintering, while the target cubic phase structure was retained under MAUST sintering. This shows that this technology can better maintain the original crystal structure of the ceramic electrolyte. Yu et al. successfully synthesized LATP powder using a microwave‐assisted hydrothermal method. [132] The LATP synthesis time was greatly shortened from 24 h to 30 min. Under the optimal conditions, the sample had high crystallinity, high density (97.2%), and high ionic conductivity (1.424 × 10^−4^ S cm^−1^) (Figure 9b). Gu et al. prepared oxygen‐deficient LATP ceramic materials by a hydrothermal method combined with high‐temperature calcination. When the calcination temperature of LATP was set to 700°C, it was found that the solid composite electrolyte composed of defect‐rich O‐LATP‐PAN had the highest ionic conductivity. When operating in the voltage range of 2.5–4.2 V and the current density of 0.1 C, the reversible discharge capacity was 220.05 mA h g^−1^. After completing 90 cycles, the battery capacity retention rate was as high as 99.74%.

In parallel, a secondary phase forms during NASICON synthesis, although it often leads to degraded ionic conductivity and interfacial instability. However, recent studies have shown that certain secondary phases can play a beneficial role during the sintering process. Luo et al. utilized (NH_4_PO_3_)_n_ as a phosphorus source and successfully synthesized a NASICON‐type LATP solid electrolyte via a solid‐phase reaction method [133]. They added Li_2_CO_3_·Bi_2_O_3_ as a sintering aid to improve the sintering and ionic conductivity of the LATP solid electrolyte. At a sintering temperature of 900°C, the total ionic conductivity of the sintered sample peaked at 0.673 mS cm^−1^. As shown in the figure, LATP‐0LB ceramic particles contain LATP and LiTiOPO_4_. LiTiOPO_4_ produces a liquid phase in the 800°C–900°C range, promoting element diffusion. The shrinkage peaks at 835.3°C and 943.9°C are likely related to liquid‐phase sintering caused by the secondary phases LiTiOPO_4_, BiPO_4_, and LiBiO_2_. This promotes liquid‐phase sintering of LATP by generating secondary phases with lower melting points (BiPO_4_ and LiBiO_2_) (Figure 9c). Using Na_2_SiO_3_ (melting point 1088°C) as a sintering additive, Oh et al. sintered NZSP at 1175°C for 10 h with a ramp rate of 5°C min^−1^ up to 950°C, then ramped to 1175°C at a rate of 1°C min^−1^ and then cooled down in a furnace [51]. The addition of Na_2_SiO_3_ promotes the densification and yields a silicon‐rich secondary phase along the grain boundaries, increasing the ionic conductivity of NZSP from 0.64 to 1.45 S cm^−1^ (Figure 9d).

#### 5.1.2 Sodium NASICON Conductor

**FIGURE 10:**
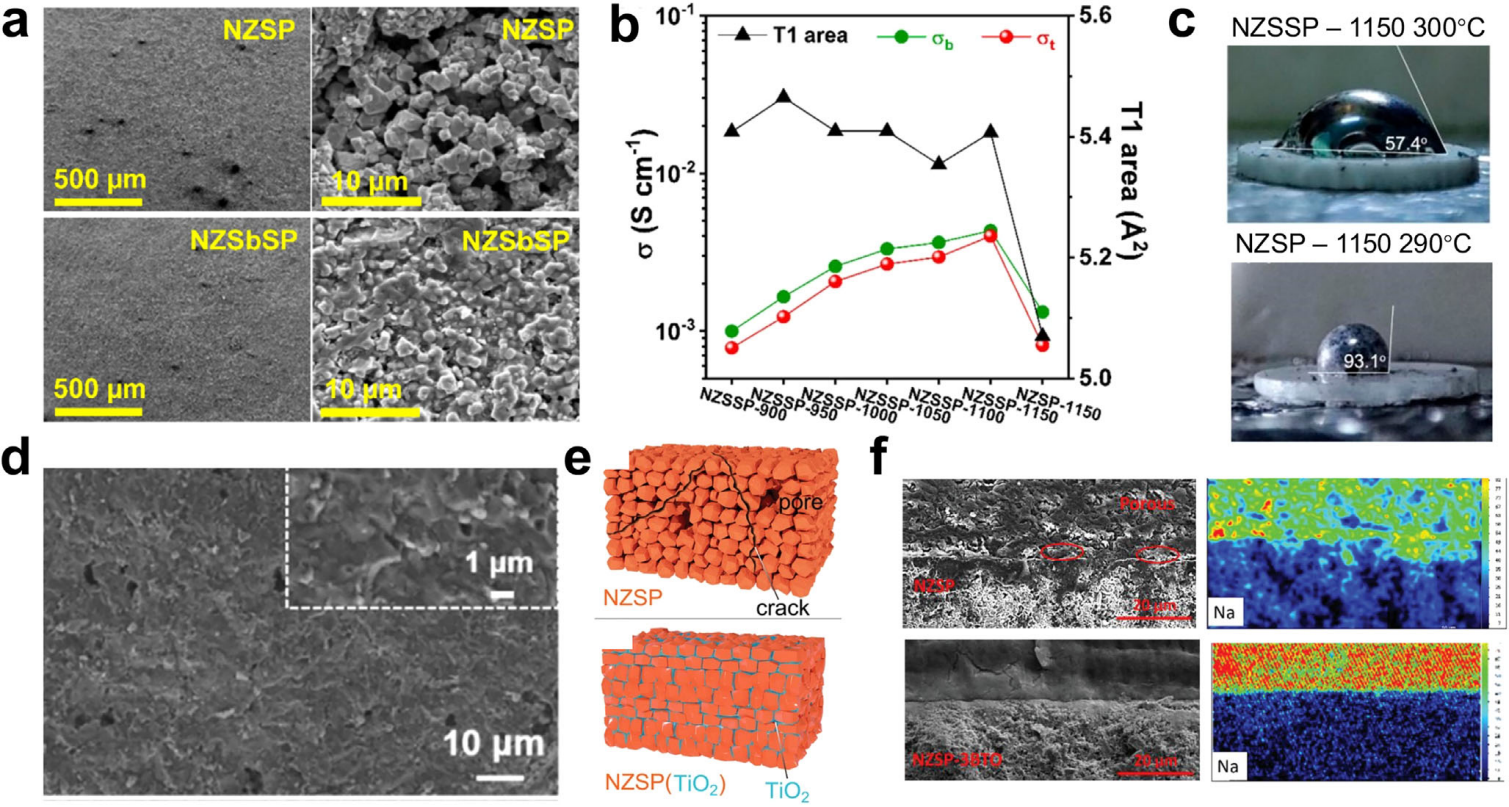
Elemental doping strategies for enhancing Na^+^ NASICON conductors. (a) SEM images of the undoped NZSP pallets with pores on the surface and doped NZSbSP with compact and densely packed particles. Reprinted with permission from Akbar et al. [46] Copyright (2025) Elsevier. (b) Bulk (σ_b_) and total (σ_t_) electrical conductivity, along with the T1 bottleneck area of the NASICON electrolytes. Reprinted with permission from Liu et al. [134] Copyright (2023) American Chemical Society. (c) Pictures of Na on NZSSP – 1150 pellets with 10ScSZ precursor at 300°C and NZSP – 1150 at 290°C. Reprinted with permission from Liu et al. [134] Copyright (2023) American Chemical Society. (d) Cross‐sectional SEM of the nominal composition NZSP‐SX (X=30) solid electrolytes. Reprinted with permission from Wang et al. [135] Copyright (2024) American Chemical Society. (e) Schematic structure of NZSP synthesized by the conventional solid‐state sintering method, showing cracks and pores, and the NZSP (TiO_2_) material, showing the second TiO_2_ phase distributed at NZSP grain boundaries and particle surfaces. Reprinted with permission from Gao et al. [106] Copyright (2022) John Wiley and Sons. (f) SEM images and EDS mapping results of metallic Na recovered from cycled Na/NZSP‐3BTO/Na and Na/NZSP‐3BTO/Na cells at 0.1 mA cm^−2^. Reprinted with permission from Sun et al. [136] Copyright (2022) John Wiley and Sons.

Similarly to Li‐based NASICON, in Na‐based NASICON, Zr^4+^ ions in NSZP are considered unstable when in contact with Na metal. Akbar et al. synthesized Sb‐doped NZSP [46]. Doping the Zr^4+^ sites of NZSP with trivalent Sb^3+^ increases the relative density, thus improving solid electrolyte ionic conductivity (Figure 10a). Experimental results show that the Sb^3+^‐doped NSZP sintered at 1200°C has an ionic conductivity value of 0.51mS cm^−1^ (Figure 10b). With this design, the symmetric battery cell maintains stable cycling for up to 465 h. The excellent cycling performance is attributed to the high surface density caused by doping and the uniform Na^+^ flux at the interface, which has an inhibitory effect on Na dendrites. Liu et al. synthesized Na_3.36_Zr_1.64_Sc_0.36_Si_2_PO_12_ (NZSSP) and simultaneously reduced the interfacial impedance through this doping strategy. The rhombohedral phase content was as high as 97.2% [134]. NZSSP‐1150 achieved a conductivity of 3.09 mS cm^−1^. Additionally, the increased content of the rhombohedral phase contributes to a better adhesion behavior, thus reducing the interfacial impedance. A contact angle was only 57.4° at 300°C, when contacting with Na metal (Figure 10c). Similarly, Wang et al. obtained Na_3.2_Zr_1.8_Sm_0.2_Si_2_PO_12_ by doping NZSP with Sm^3+^, and the total conductivity of the electrolyte was 1.87 mS cm^−1^ at 23°C (Figure 10d) [135].

In addition to pure doping and the beneficial secondary phases that can arise as a side effect, artificially introducing secondary phases can also play a critical role in suppressing Na dendrites [78]. Gao et al. added anatase to the precursor and introduced TiO_2_ as a secondary phase to form within grain boundaries to mitigate microcracks and thus suppress dendrite penetration. (Figure 10e) [106] By doing so, the Na|NZSP (TiO_2_)|Na battery cell cycled for 750 h at a current density of 0.1 mA cm^−2^ without shorting behavior. Sun et al. introduced an interesting ferroelectric phase BaTiO_3_ (BTO) into NZSP to help establish an internal electric field and achieve uniform deposition/stripping of Na at the Na/solid‐electrolyte interface [136]. This is achieved by spontaneous polarization induced by the ferroelectric phase (Figure 10f). The ferroelectric phase can not only bridge and deflect the cracks, but also consume the driving force of crack propagation. The mechanical energy generated by the crack can be converted into electrical energy through the piezoelectric effect, or simultaneously consumed by the stress‐induced ferroelectric phase transition. Therefore, the ferroelectric phase can also alleviate the stress caused by the high volume change of the Na anode and ensure the integrity of the interface. In addition to the spontaneous polarization effect, this secondary phase also suppressed Na metal dendrite by improving electrolyte densification and interfacial contact with the Na anode. The Na|NZSP‐3BTO|Na battery exhibited stable cycling performance for up to 1000 h at 0.1 – 0.3 mA cm^−2^. Even when the current density was increased to 0.3 mA cm^−2^, there was still no obvious short circuit, and a long‐term cycling of more than 1000 h was achieved with an overpotential of 60 mV.

**FIGURE 11:**
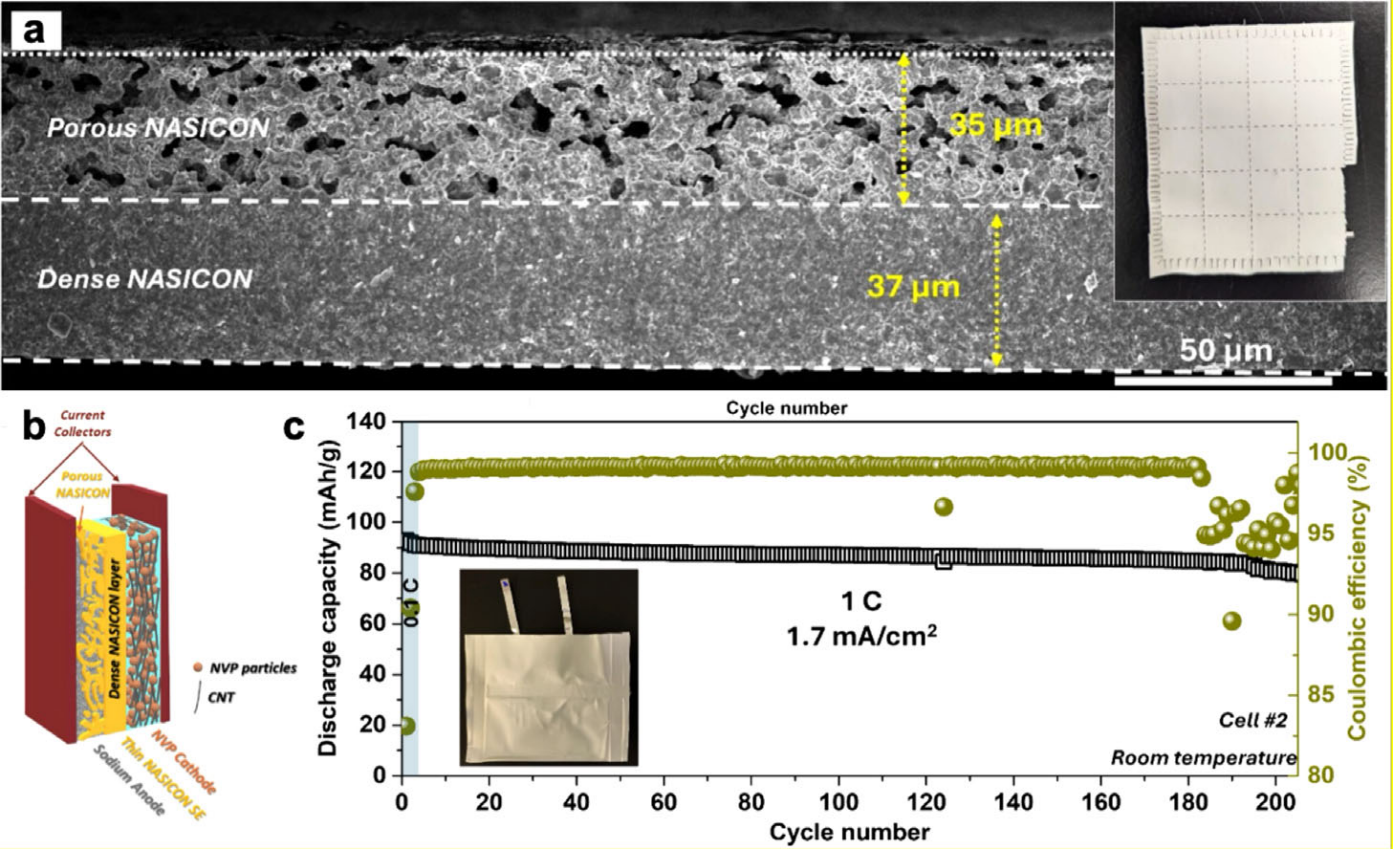
Bilayer‐structured and dual‐doped NASICON electrolytes for high‐rate and stable sodium metal batteries. (a) Cross‐sectional SEM image of a bilayer NASICON electrolyte (inset: digital photograph of 5 × 4 batches of 1 cm^2^ bilayer membranes) [138]. (b) A schematic of Na solid‐state pouch cell [138]. (c) long‐term cycling performance of the Na‐metal‐infiltrated NASICON bilayer with the NVP cathode active material. Figures a–c are reproduced from the same reference, and reprinted with permission from Jaschin et al. [138] Copyright (2025) American Chemical Society.

Apart from chemically driven approaches discussed all above, constructing a 3D electrolyte structure is a very effective strategy to mitigate metal dendrite formation. Jaschin et al. developed a dual‐doped (i.e., Mg^2+^ and Zn^2+^) Na_3+2 (x+y)_Zr _2− (x+y)_Zn_x_Mg_y_Si_2_PO_12_ (x Zn = 0.2 and y Mg = 0.125) trilayer structure (i.e., porous‐dense‐porous layer) [137]. With a ZnO atomic layer deposition, such trilayer Na‐based NASICON demonstrated stable voltage hysteresis at current densities of 5, 10, and 15 mA cm^−2^ over a total cycling time of 620 h. This 3D structure dramatically enhanced the contact area between the metal electrode and the solid electrolyte, thus sharply reducing the localized current density and subsequently preventing metal dendrite formation. Given the generalization of this strategy, it can also be applied to Li‐based NASICON. Jaschin et al. also synthesized a Zn‐ and Mg‐doped NASICON electrolyte separator layer within a 3D porous bilayer structure (Figure 11a). Using Na metal as the anode and sodium vanadium phosphate (NVP) as the cathode active material, resulting in a full cell configuration shown in Figure 11b. The first two charge–discharge cycles were performed at 0.1 C, followed by over 200 cycles at 1 C. At 1 C, the initial discharge capacity was 91 mAh g^−1^. Furthermore, the capacity retention was 89% at the end of 200 cycles. The cell also exhibited a stable Coulombic efficiency of 99% for 180 cycles (Figure 11c).

### 5.2 Metal Anode Design

Metal electrode modification is also a very effective strategy for addressing the NASICON/metal interface problem. This is achieved by improving interfacial contact, thereby reducing void formation. The strategies include introducing alloying, composite electrodes, a 3D metal host, and stack pressure.

**FIGURE 12:**
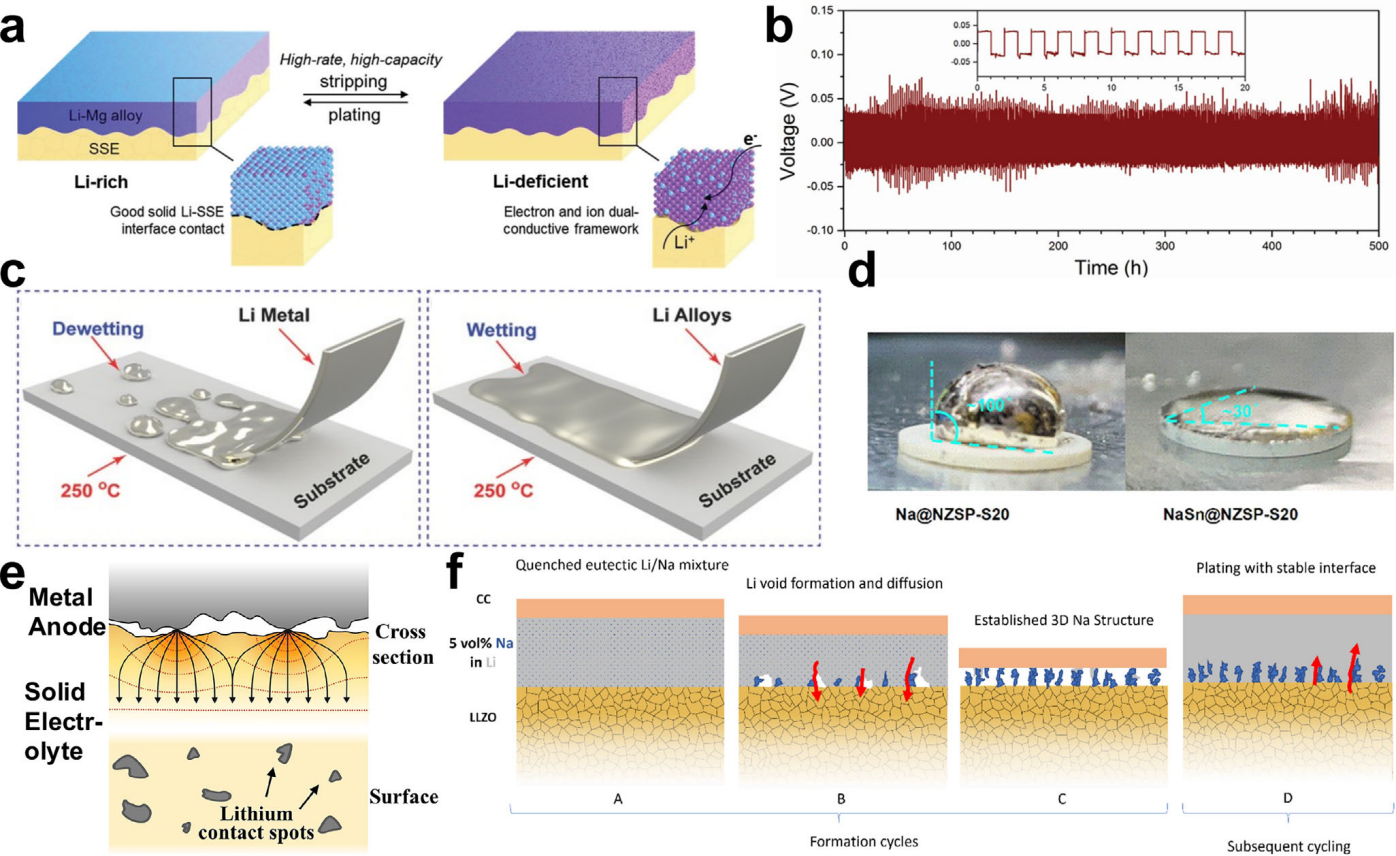
Interface contact and wetting strategies for metal anodes in solid‐state batteries. (a) Schematics of the Li stripping/plating at high rate and high capacity in solid‐state cells with a garnet‐type solid electrolyte, using a pure Li‐metal anode and the Li–Mg alloy anode. Reprinted with permission from Yang et al. [141] Copyright (2018) John Wiley and Sons. (b) Electrochemical performance of the Li–Mg alloy anode. The cell cycled at 1 mA cm^−2^ for 1 h in each half cycle for a total of 500 h. Reprinted with permission from Yang et al. [141] Copyright (2018) John Wiley and Sons. (c) Schematic of soldering Li and Li alloy onto substrates (e.g., ceramics, polymers, and metals). Reprinted with permission from Wang et al. [142] Copyright (2017) John Wiley and Sons. (d) Contact angle measurements of molten Na (left) and the NaSn alloy (right) on NZSP‐S20. Reprinted with permission from Wang et al. [135] Copyright (2024) American Chemical Society. (e) Schematic representation of the metal/oxide solid electrolyte interface showing a few contact points as the origin of constriction and the resultant bending of the current lines at the interface, which is the basis of the applied theory. v At higher external forces, the contact spots increase in area due to the plastic deformation of the softer material (i.e., Li). The arrows schematically indicate current lines. Equipotential lines are shown as red dotted lines. Reprinted with permission from Krauskopf et al. [145] Copyright (2019) American Chemical Society. (f) Schematic of the formation mechanism of the 3D Na interface structure, starting with the monotectic mixture after quenching (A), Li migration through the bulk and along void surfaces (red arrows) accompanied by consolidation of the metal electrode during stripping (B), the fully established 3D Na structure in the Li depleted state after high‐capacity stripping (C) and the most likely metal electrode structure upon replating of the full Li capacity (D). Reprinted with permission from Mann et al. [146] Copyright (2025) Elsevier.

Through metal alloying: in Li‐based NASICON, Li can be alloyed with metals such as Al, Mg, Zn, Ag, and Bi. These alloy anodes offer multiple advantages over pure Li, including higher electrochemical potential, higher Li‐ion diffusion coefficients, and improved wettability with solid electrolytes, collectively contributing to enhanced interfacial stability [139, 140]. Yang et al. replaced the pure Li metal anode with a Li‐Mg (20 wt% of Mg) alloy [141]. The Li‐Mg alloy exhibited excellent wettability (Figure 12a). With such anode, the Li‐Li symmetric cells demonstrated stable cycling performance at a current density of 1 mA cm^−2^, lasting 250 cycles (Figure 12b). Similarly, Wang et al. found that by using Li‐Sn alloy (20–50 wt% Sn), the oxide solid electrolyte (e.g., garnet solid electrolyte) was wetted by the alloy within 10 s (Figure 12c) [142]. In Na‐based NASICON, Wang et al. used a Na‐Sn alloy to improve the wetting ability between the metal‐based electrode and the solid electrolyte [135]. The contact angle of Na‐Sn alloy (20–50 wt% Sn) on the Na_3.2_Zr_1.8_Sm_0.2_Si_2_–PO_12_ surface is only ∼30°, which effectively prevents uneven contact between the electrode and the electrolyte and reduces metal dendrite formation (Figure 12d). Whereas, the contact angle of pure molten Na on the surface of Na_3.2_Zr_1.8_Sm_0.2_Si_2_–PO_12_ reached 100°.

In addition to binary metal alloys, metal electrodes can also be compounded with non‐metallic and metal compounds to form composite electrodes, which help mitigate metal dendrite formation [100]. Lu et al. prepared a thin‐layer Na‐Ti_3_C_2_T_x_ composite anode and used a PVDF‐HFP‐Na_3_Zr_2_Si_2_PO_12_‐modified polyimide separator (M‐PI) as the electrolyte [143]. The performance of the negative electrode and electrolyte was further evaluated using a Na‐Ti_3_C_2_T_x_|M‐PI|Na‐Ti_3_C_2_T_x_ symmetric cell. At a current density of 0.5 C, the charge–discharge capacity reached 89.7 mAh g^−1^ after 400 cycles. Chen et al. [144] used metal‐organic frameworks as derived materials, nitrogen‐doped porous carbon (NCC) as a carrier, and embedded cobalt particles as a 3D matrix to construct an ultrathin sodium anode on the surface of NASICON electrolyte. The 3D mixed ion‐electron conductor (MIEC) within this composite anode effectively promoted sodium ion transport, achieving dendrite‐free sodium deposition. A symmetric cell using 30 µm NCC‐Na as the anode could operate stably for over 6000 h at a current density of 0.2 mA cm^−2^.

As evident above, both strategies are beneficial for interfacial void closure. At a fundamental level, the primary cause of void formation at the rigid NASICON/metal interface is the low self‐diffusion coefficient of the Li/Na metals. In addition to a chemically driven approach, mechanically tuning the battery cell stack pressure during operation is also an effective method to achieve good contact between the metal anode and the solid electrolyte. For example, Krauskopf et al. demonstrated that the interfacial resistance between a metal anode and an oxide solid electrolyte was minimized when the pressure was increased to 400 MPa and then relaxed to standard pressure (Figure 12e) [145].

Sufficient stack pressure (i.e., above the yielding strength of the metal anode) causes plastic deformation, reducing the potential for void formation at the interface. However, adding a stack pressure introduces additional cost to battery operation. Thus, an alternative approach to compensate in this regard is to increase the effective contact area through the concept of a 3D metal anode. Mann et al. mechanically mixed a small amount of Na into Li metal (LiNa) before anode fabrication, resulting in the in situ formation of a 3D sodium scaffold within the Li matrix. (Figure 12f) [146] Symmetric LiNa|oxide‐solid‐electrolyte|LiNa cells delivered a total capacity of 1 mAh cm^−2^ at 0.5 mA cm^−2^ and 5 mAh cm^−2^ at 0.1 mA cm^−2^ at 60°C. In both cases, pure Li metal 2D anodes with unoptimized interfaces resulted in increased interfacial resistance, ultimately leading to dendrite formation. Similarly, Shi et al. fabricated a Cu mesh coated with an Ag layer (ACM) and soldered it to the surface of an oxide solid electrolyte [147]. Molten metal was then wetted and spread onto the solid electrolyte surface at 900°C with the aid of the ceramic‐mounted ACM, significantly reducing the interfacial resistance from 2869.05 to 34.1 Ω cm^2^. The Li/100ACM‐solid electrolyte/Li symmetric cell exhibited excellent stability after cycling for 800 h at a current density of 0.1 mA cm^−2^.

### 5.3 Solid Electrolyte / Metal Interface Design

By introducing a ‘third‐party’ protective layer, the interface problem between NASICON electrolyte and the metal electrode can be effectively improved. Poor wetting, mechanical mismatch, and the formation of interfacial reaction layers contribute to increased interfacial resistance, dendrite formation, and eventual battery failure. To address these issues, researchers have developed a wide array of interface engineering strategies that fall into three main categories: (i) introducing trace liquids to enhance wettability and ion transport; (ii) constructing ion‐conductive solid interlayers to block electronic leakage while facilitating uniform lithium/sodium ion flux; and (iii) designing mixed ionic‐electronic conductor (MIEC) layers to redistribute electric fields and suppress dendritic growth. These strategies have been successfully applied in both Li^+^ and Na^+^ NASICON systems. The following subsections summarize representative advances in Li‐ and Na‐based systems, highlighting how interface optimization enables more robust and high‐performance NASICON‐based ASSBs.

#### 5.3.1 Lithium‐Based System

Poor solid‐solid interface contact between the electrode and the Li^+^ NASICON severely limits the electrochemical performance of the battery. To overcome this interfacial problem, introducing a small amount of liquid into the gap between the solid electrolyte and the electrode is considered a convenient and effective strategy. Ionic liquids (ILs) have emerged as promising candidates as wetting agents at the solid electrolyte interface due to their non‐volatility, non‐flammability, and high ionic conductivity. For example, Reinoso et al. infiltrated an IL solution (i.e., Pyr_14_TFSI‐based IL) into sintered LATP ion‐conducting porous ceramics, improving the ionic conductivity to 10^−3^ at 30°C [148]. The electrochemical performance of LATP‐0CS‐IL and LATP‐5CS‐IL electrolytes (500 µm thickness) was tested in CR2032 batteries with lithium iron phosphate (LFP) and lithium (Li) as the positive and negative electrodes, respectively, at 20°C. The results show that the capacity is as high as 150 mAh g^−1^ LFP at C/30 rate and 60 mAh g^−1^ LFP can still be maintained at 1 C rate, while pure LATP performs poorly at low temperatures.

**FIGURE 13:**
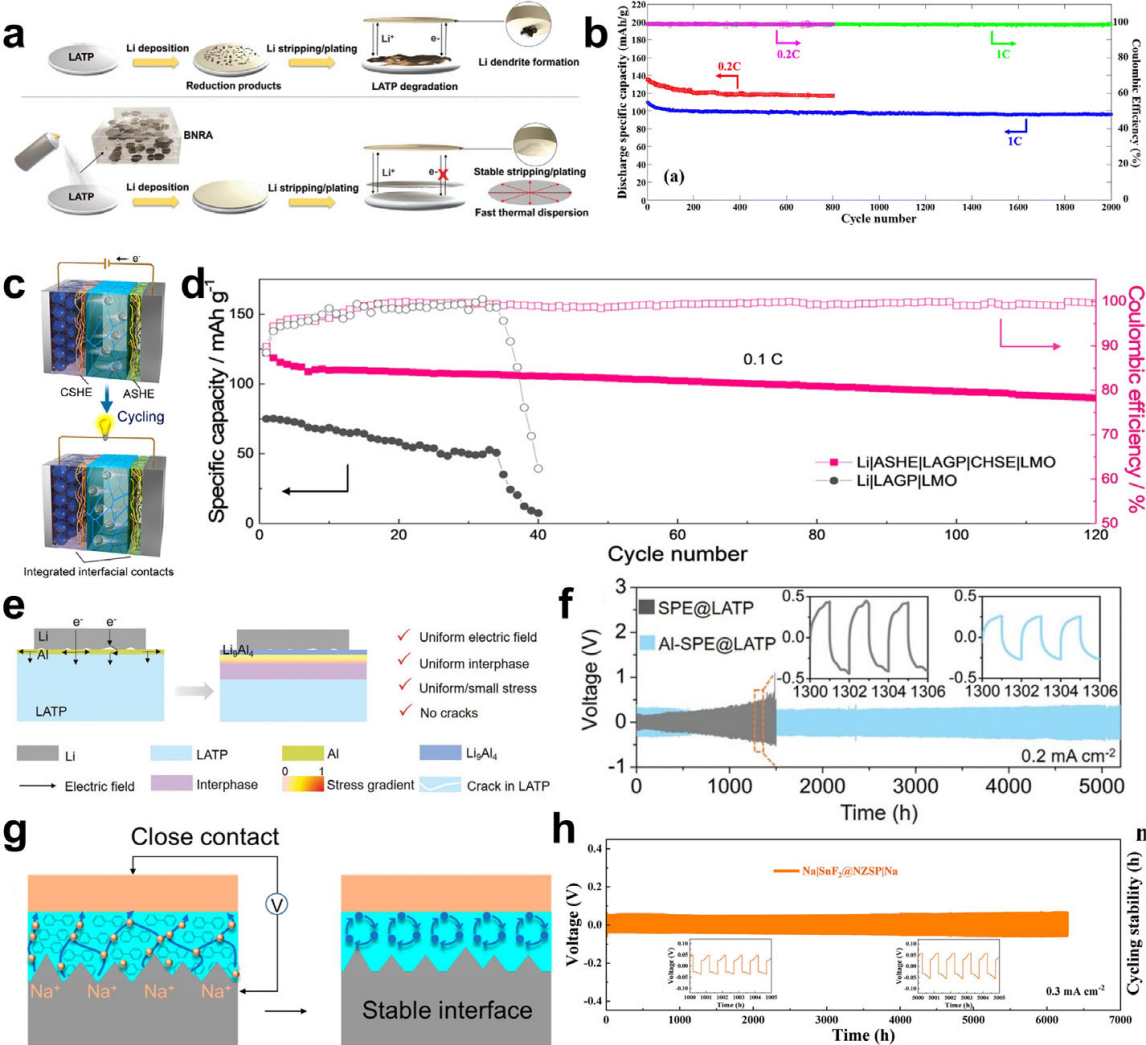
Representative interface design strategies at the solid electrolyte/metal interface and their electrochemical performance. (a) Schematic diagram of preparations LATP pellets with and without BNRA modification, and the function mechanism of BNRA. Reprinted with permission from Zhu et al. [149] Copyright (2022) John Wiley and Sons. (b) Long‐term electrochemical performances (coulombic efficiency and discharge specific capacity versus cycle number) of the Li|CPCE|LFP cells at 0.2 and 1 C, respectively. Reprinted with permission from Ghafari et al. [150] Copyright (2023) Royal Society of Chemistry (c) Schematic illustrations of the interfacial evolution in Li|LAGP|LMO batteries with SHEs as Janus interface layers during cycling. Reprinted with permission from Liu et al. [151] Copyright (2020) American Chemical Society. (d) Cycling performances of Li|LAGP|LMO and Li|ASHE|LAGP|CSHE|LMO cells at 0.1 C. Reprinted with permission from Liu et al. [151] Copyright (2020) American Chemical Society. (e) The interface evolution schematics of Li/LATP (with Al interlayer). Reprinted with permission from Luo et al. [152] Copyright (2023) John Wiley and Sons. (f) Voltage profiles of Li/Li symmetric cells with SPE interlayer or Al‐SPE interlayer at 0.2 mA cm^−2^ and 50°C, inset is voltage profiles from 1300 to 1306 h. Reprinted with permission from Luo et al. [152] Copyright (2023) John Wiley and Sons. (g) Designed a stable liquid film interface. The close contact and the rapid charge transfer at the interface could ensure the excellent long‐term stability and rate performance. Reprinted with permission from Meng et al. [47] Copyright (2022) American Chemical Society. (h) The cycling stability of the Na|SnF_2_@NZSP|Na cell at 0.3 mA cm^−2^ at 25°C. Reprinted with permission from Yang et al. [154] Copyright (2023) Elsevier.

In addition to a liquid‐based interlayer, constructing an ion‐conductive solid interlayer that can hinder the interfacial side reaction is also very promising. Zhu et al. used a commercial boron nitride‐based release agent (BNRA) slurry as a coating material for LATP (Figure 13a) [149]. The N atoms in BNRA are highly lithiophilic and can form Li‐N in situ at the Li/BNRA‐LATP interface, thus forming a stable Li^+^ NASICON conductive medium between the LATP electrolyte and the Li metal, achieving uniform Li deposition‐stripping. The overpotential of the Li|BNRA‐LATP|Li cell increases to only ±66 mV after cycling for more than 1200 h at 0.2 mA cm^−2^ and remains stable. Stable lithium stripping‐deposition performance is achieved even at a critical current density (CCD) of 1 mA cm^−2^. In parallel, Ghafari et al. developed a new protective layer (PL) composed of a PVDF‐HFP polymer matrix and LiF ceramic particles [150]. This new composite ceramic polymer electrolyte (CPCE), in which LATP particles are sandwiched between two gel polymer electrolytes (GPEs), has the structural formula PL|LATP|PL. This composite protective layer has extremely low electronic conductivity (3 × 10^−10^ S cm^−1^) and high ionic conductivity (0.59 mS cm^−1^), as well as extremely high mechanical strength, with a modulus of up to 55 GPa. The Li|CPCE|Li symmetric cell was electroplated/stripped stably for 1000 h at a current density of 0.5 mA cm^−2^ (Figure 13b). The same ion conductor strategy should also work well in other Li‐based NASICON (e.g., LAGP). Liu et al. developed a self‐healing polymer electrolyte (SHE) as an ion conductor layer to protect LAGP (Figure 13c) [151]. The SHE has high flame retardancy and exhibits high ionic conductivity of about 10^−3^ S cm^−1^ at 25°C, inhibiting side reactions and suppressing the formation of dendrites. The Li|ASHE|LAGP|ASHE|Li cell exhibits voltage hysteresis with no obvious oscillations during 700 h of cycling, and the overpotential (∼200 mV) is much lower than that of the cell using AGPE without self‐healing segments as the interfacial layer (Figure 13d).

Physically separating the metal electrode and solid electrolyte through an ionic‐conductive layer works very well. Equally important, adding electronic conduction to form a mixed Ionic‐Electronic conductor (MIEC) interlayer is also a popular approach used in solid electrolytes to improve electrode/electrolyte interface stability due to reduced interfacial overpotential. Luo et al. found in their experiments that Al or Ag interlayers can greatly improve the interfacial stability of Li/LATP [152]. For bare LATP, the uneven electric field distribution at the Li/LATP interface leads to uneven growth of the reacted LATP interphase during cycling (Figure 13e). This promotes stress concentration at the edge region between the interphase and the unreacted LATP, leading to continuous crack propagation. In contrast, Al/Li_9_Al_4_ exhibits excellent electronic conductivity, and the Al‐modified LATP (Al@LATP) facilitates a more uniform electric field distribution. This uniformity promotes homogeneous interphase growth, thereby relieving local stress and mitigating mechanical degradation at the interface. Although the Al interlayer alone can enhance the interfacial stability of Li/LATP, it still has difficulty in suppressing the injection of electrons and protecting LATP from reduction. Liu et al. introduced Li_4_Ti_5_O_12_ (LTO) as an interfacial protective layer [153]. LTO not only effectively slows down the side reactions between Li metal and LATP, but also acts as a medium for Li^+^ NASICON diffusion at the interface. In addition, when LTO is in direct contact with the Li metal, LTO undergoes lithiation, introducing electronic conductivity while maintaining ionic conductivity. Experimental results show that the Li|LATP@LTO|Li symmetric battery cell achieves a stable cycle of 3500 h at 0.2 mA cm^−2^, and can maintain a stable cycle of 1000 h even at 0.5 mA cm^−2^ (Figure 13f).

#### 5.3.2 Sodium‐Based System

Similarly, as Li‐based NASICON, the wettability of NZSP solid electrolytes with Na metal anode is relatively poor. Additionally, the interfacial decomposition compounds, including Na_4_SiO_4_, Na_2_ZrO_3,_ Na_2_CO_3_ and NaOH further slowed down the Na^+^ transport at the NZSP/Na metal interface. Therefore, interface modification is also necessary for stabilizing the Na metal and solid electrolyte interface. This section is constructed in parallel with Section 5.3.1.

Meng et al. constructed a new liquid Na‐biphenyl‐based interphase to regulate Na metal and Na_3_Hf_2_Si_2_PO_12_ (NHSP) [47]. The symmetric battery cell designed with this interphase was stably cycled for more than 1000 and 700 h at current densities of 0.2 and 0.5 mA cm^−2^, respectively at room temperature.

**FIGURE 14:**
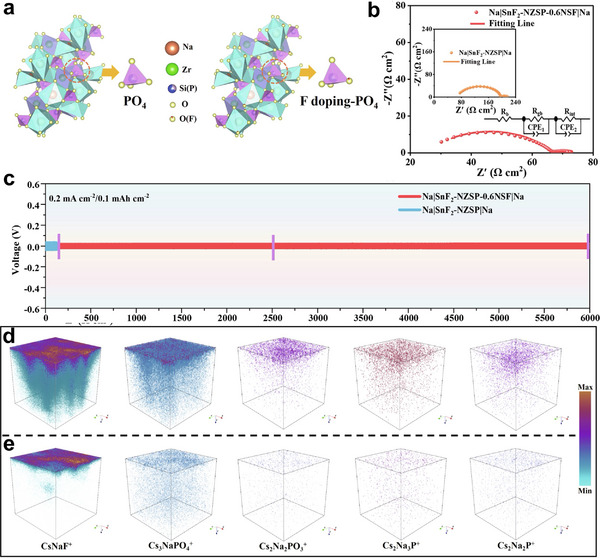
Enhanced PO covalency in NZSP and SnF_2_ interface regulation to synergistically stabilize the Na|NZSP interface and improve long‐term cycling performance. (a) Schematic of crystal structures of NZSP and NZSP‐0.6NSF after NPD Rietveld refinement (the orange balls represent Na atoms, green balls represent Zr atoms, blue and purple balls represent Si and P atoms, respectively, yellow balls represent O atoms, and red balls represent F atoms). Reprinted with permission from Li et al. [100] Copyright (2025) Springer Nature. (b)The symmetric cell impedance spectra for NZSP‐0.6NSF and NZSP (inset). Reprinted with permission from Li et al. [100] Copyright (2025) Springer Nature. (c) Galvanostatic Na plating/stripping performance of the Na|SnF_2_‐NZSP|Na and Na|SnF_2_‐NZSP‐0.6NSF|Na symmetric cell under 0.2 mA cm^−2^/0.1 mAh cm^−2^ at 30°C. Reprinted with permission from Li et al. [100] Copyright (2025) Springer Nature. (d,e) The 3D render TOF‐SIMS of CsNaF^+^, Cs_3_NaPO_4_^+^, Cs_2_Na_2_PO_3_^+^, Cs_2_Na_3_P^+^, and Cs_2_Na_2_P^+^ of cycled‐NZSP and cycled‐NZSP‐0.6NSF for Na+SnF_2_|NZSP‐0.6|Na+SnF_2_ and Na+SnF_2_|NZSP|Na+SnF_2_ (The cyan represents low concentration of the substance, while brown represents high concentration of the substance). Reprinted with permission from Li et al. [100] Copyright (2025) Springer Nature.

Li et al. achieved high‐rate performance and a stable quasi‐SSB by introducing Na_2_SiF_6_ (NSF) to enhance the PO covalency in NZSP and applying a SnF_2_ coating to establish a stable interface [100]. NZSP‐xNSF (*x* = 0, 0.2, 0.4, 0.6, 0.8, and 1.0 wt%) were synthesized using a facile high‐temperature solid‐phase method (Figure 14a). NZSP‐0.6NSF exhibited a leading ionic conductivity of 1.3 × 10^−3^ S cm^−1^. To compare the battery performance of NZSP and NZSP‐0.6NSF, the interface was modified with SnF_2_. NZSP‐0.6NSF exhibited improved structural stability, preventing reaction with SnF_2_ at high temperatures, which would otherwise destroy the NZSP structure. The interfacial impedances of the Na|SnF_2_‐NZSP‐0.6NSF|Na and Na| SnF_2_‐NZSP|Na symmetric cells were 2.0 and 6.5 Ω cm^2^, respectively (Figure 14b). This indicates that NZSP‐0.6NSF can form a more stable interface with SnF for Na^+^ transport. The Na| SnF_2_‐NZSP‐0.6NSF|Na symmetric half‐cell operated stably for 6000 h at a current density of 0.2 mA cm^2^ with a polarization voltage of only 15 mV (Figure 14c). DFT calculations were performed at the Na|NZSP interface, revealing that when the PO tetrahedrons of the NZSP interact with Na atoms, the PO bonds within the PO tetrahedrons break, forming Na_x_PO_y_ or Na_x_P, leading to rapid decomposition of the solid electrolytes. In contrast, the F‐PO tetrahedrons of the NZSP‐NSF maintain an intact structure at the Na|NZSP‐NSF interface and do not disintegrate. This indicates that the presence of F atoms can effectively inhibit the destruction of the PO bond by metallic Na, and the structural difference between the Na|NZSP and Na|NZSP‐NSF interfaces is mainly attributed to the changes in the electronic structure and covalency of the PO bond in NZSP caused by the doping of F atoms (Figure 14d,e).

As with the previous discussion of the Li‐ion system, preparing a MIEC layer with a uniform Na^+^ flux and electric field distribution would be a great choice. Yang et al. established a Na_15_Sn_4_@NaF mixed ion/electronic conductive layer [154]. NaF has high ionic conductivity and a low room temperature diffusion energy barrier, and its electronic insulating properties can further inhibit the growth of Na dendrites (Figure 13g). The Na/Sn alloying reaction enables NZSP to exhibit excellent wettability to Na metal, significantly reducing interfacial resistance and enhancing the stability of the interface during cycling. The assembled symmetric sodium battery exhibits an extremely low interfacial impedance of 2 Ω cm^2^ and a high critical current density of 1.3 mA cm^−2^ at 25°C. Even at a high current density of 0.3 mA cm^−2^, the symmetric sodium battery exhibits excellent cycling performance of over 6000 h (Figure 13h).

To better clarify the effectiveness of various interface engineering strategies applied to NASICON‐type solid electrolytes, we summarize in Table 1 the key electrochemical properties achieved through representative approaches, including surface coatings, composite interlayers, dopant modifications, and metal alloying. Specifically, we highlight the improvements in ionic conductivity, interfacial resistance, critical current density (CCD), cycling stability, and electrochemical oxidation stability as these parameters collectively govern the performance and reliability of NASICON‐based solid‐state batteries. This comparative summary provides a concise reference for evaluating the practical potential and design trade‐offs of each strategy, offering insights into their applicability across different NASICON systems.

**TABLE 1:** Summary of NASICON property and strategies to stabilize with metal anode.

Chemical formula	Strategy (bulk and interface)	Ionic conductivity (S cm^−1^)	Interface Resistance (Ω cm^2^)^*^	CCD (mA cm^−2^)	Full Cell Performance		Oxidation Stability^****^	Refs.
					Cathode Active Material^**^	Cycling Performance^***^		
Li_1.6_Al_0.4_Mg_0.1_Ge_1.5_ (PO_4_)_3_	Bulk: Mg doping	7.4 × 10^−3^	—	2	LiNi_0.8_Mn_0.1_Co_0.1_O_2_,N/A	0.2 mA cm^−2^, 156.5 mAh g^−1^, 250 cycles	2.4–4.3 V Li/Li^+^	[129]
Li_1.5_Al_0.5_Te_x_Ge_1.5‐x_ (PO_4_)_3_	Bulk: Te doping	6.33 × 10^−4^	—	0.5	LiFePO_4_, N/A	0.1 mA cm^−2^, 145mAh g^−1^, 100 cycles	4.0 V vs. Li/Li^+^	[130]
Li_1.3_Al_0.3_Ti_1.7_ (PO_4_)_3_	Bulk: commercial boron nitride‐based release agent (BNRA) slurry	1.69 × 10^−4^	—	1	LiFePO_4_, 1.1 mg cm^−2^	0.5 C, 151.6 mAh g^−1^, 500 cycles	3.6 V vs. Li/Li^+^	[149]
Li_1.5_Al_0.5_Ge_1.5_P_3_O_12_	Interface: self‐healing polymer electrolytes (SHEs) as Janus interfaces	10^−3^	—	1	LiMn_2_O_4_, 1.2 mg cm^−2^	0.1 C, 101.3 mAh g^−1^, 120 cycles	4.7 V vs. Li/Li^+^	[151]
Li_1.3_Al_0.3_Ti_1.7_ (PO_4_)_3_	Bulk: an atomic welding strategy bridged by Ni doping	3.77 × 10^−4^	248	2	LiFePO_4_, 1.7 mg cm^−2^	0.5 C, 150.3 mAh g^−1^, 200 cycles	3.5 V vs. Li/Li^+^	[49]
Li_1.3_Al_0.3_Ti_1.7_ (PO_4_)_3_	Interface: a Li metal–ceramic composite (LMCC) foil anode (incorporating Li_6.4_La_3_Zr_1.4_Ta_0.6_O_12_ into Li metal)	2.36 × 10^−4^	—	0.8	LiFePO_4_, 1.11‐1.84 mg cm^−2^	1.88 C, 76.5 mAh g^−1^, 500 cycles	4 V vs. Li/Li^+^	[155]
Li_1.5_Al_0.5_Ge_1.5_P_3_O_12_	Interface: C_3_N_4_	8.96 × 10^−4^	—	2	LiFePO_4_, N/A	0.1 C, 143.5 mAh g^−1^, 300 cycles	2.5–4.2 V vs. Li/Li^+^	[156]
Li_1.5_Al_0.5_Ge_1.5_P_3_O_12_	Interface: Pt@LAGP	1.3 × 10^−4^	141	0.6	LiNi_1/3_Co_1/3_Mn_1/3_O_2_, N/A	0.1 C, 133.5 mAh g^−1^, 100 cycles	2.4–4.2 V vs. Li/Li^+^	[157]
Li_1.5_Al_0.5_Ge_1.5_P_3_O_12_	Interface: a composite solid electrolyte (CSE) composed of poly (ethylene oxide) (PEO) and LATP@SI	1.15 × 10^−4^		0.3	LiFePO_4_, N/A	0.2 C, 145.95 mAh g^−1^, 200 cycles	5 V vs. Li/Li^+^	[48]
Na_3.1_Zr_1.9_Sb_0.1_Si_2_PO_12_	Bulk: Sb doping	0.51 × 10^−3^	—	0.3	O3‐Na_0.99_Zn_0.22_Fe_0.3_Mn_0.48_O_2_, 1.9–2.0 mg cm^−2^	0.1 C, 99.195 mAh g^−1^, 50 cycles	6 V vs. Na/Na^+^	[46]
Na_3.36_Zr_1.64_Sc_0.36_Si_2_PO_12_	Bulk: Sc doping	3.09 × 10^−3^	4.7	0.85	Na_3_V_2_ (PO_4_)_3_, 2.0 mg cm^−2^	1 C, 103.05 mAh g^−1^, 350 cycles	2.5–3.8 V vs. Na/Na^+^	[134]
Na_3_Zr_2_Si_2_PO_12_	Bulk: Secondary phase (TiO_2_)	6.6 × 10^−4^	13.3	1	Na_3_V_2_ (PO_4_)_3_/super P/PVDF, 1.3 mg cm^−2^	0.2 C, 98.45 mAh g^−1^, 100 cycles	3.4 V vs. Na/Na^+^	[106]
Na_3_Zr_2_Si_2_PO_12_‐3BaTiO_3_	Bulk: Secondary phase (BaTiO_3_)	0.96 × 10^−3^	65.1	1.05	Na_3_V_1.5_Cr_0.5_ (PO_4_)_3_, 3 mg cm^−2^	30 mA g^−1^, 111.5 mAh g^−1^, 400 cycles	3.9 V vs. Na/Na^+^	[136]
Na_3+2 (x+y)_Zr _2− (x+y)_Zn_x_Mg_y_Si_2_PO_12_	Bulk: three‐dimensional electrolyte structure	2.7 × 10^−3^	9	40	Na_3_V_2_ (PO_4_)_3_, 2 mg cm^−2^	0.2 C, 96.28 mAh g^−1^, 300 cycles	4 V vs. Na/Na^+^	[137]
Na_3.2_Zr_1.8_Sm_0.2_Si_2_PO_12_	Bulk, but from metal anode side: NaSn alloy electrode	1.87 × 10^−3^	—	2.2	Na_3_V_2_ (PO_4_)_2_F_3_, N/A	0.1 C, 78.15 mAh g^−1^, 200 cycles	4.3 V vs. Na/Na^+^	[135]
Na_3_Hf_2_Si_2_PO_12_	Interface: liquid Na‐biphenyl‐based interphase	1.03 × 10^−3^	—	3.6	Na_3_V_2_ (PO_4_)_3_, 5 mg cm^−2^	1 C, 104.54 mAh g^−1^, 300 cycles	3.5 V vs. Na/Na^+^	[47]
Na_3_Zr_2_Si_2_PO_12_‐xNa_2_SiF_6_	Interface: SnF_2_ coating	1.3 × 10^−3^	2.0	1.3	Na_3_V_2_ (PO_4_)_3_, 1.53 mg cm^−2^	1 C, 103.425 mAh g^−1^, 2500 cycles	2.5–3.8 V vs. Na/Na^+^	[100]
Na_3_Zr_2_Si_2_PO_12_	Interface: Na_15_Sn_4_@NaF mixed ion/electronic conductive layer	0.51 × 10^−3^	2	1.3	Na_3_V_2_ (PO_4_)_3_, 1 mg cm^−2^	1 C, 103.14 mAh g^−1^, 500 cycles	3.8 V vs. Na/Na^+^	[154]

* It refers to the interfacial resistance between the solid electrolyte and the metal anode before cycling.

** It lists the cathode active material chemistry and mass loading.

*** Cycling performance summarized here is full‐cell cycling data. Specifically, the content here is written in the following format: C‐rate, averaged capacity, and the cycle numbers.

**** Although the authors did not release the oxidation stability value in these papers, the oxidation stability value is obtained using the full‐cell cycling upper voltage

## 6 NASICON Manufacturing

Having established a comprehensive understanding of the fundamental mechanisms, identified existing challenges, and developed corresponding solutions, bridging the gap between laboratory‐scale NASICON‐based solid‐state battery implementation and industrial manufacturing becomes crucial for advancing this highly applied field. In this section, we review approaches for overcoming existing challenges through advanced fabrication methods, evaluate viable techniques from an industrialization perspective, and propose feasible manufacturing strategies based on industrial assembly lines. This section primarily focuses on metal anodes and NASICON‐based solid electrolytes. We summarized the manufacturing methods that we discussed in Section 6.1 in Table 2.

**TABLE 2:** Key metrics evaluation of solid electrolytes manufacturing methods.

Process	Thickness range	Cost	Scalability	Defect control	Customizability
Slurry Casting	20–250 µm	Low	Very High	Middle	Low
Particle Spraying	15–100 µm	Middle	Middle	Low	Middle
Mixture Extrusion	100–150 µm	Very Low	High	Low	Low
Vapor Deposition	1–10 µm	High	Low	High	Very High

### 6.1 Solid Electrolytes Manufacturing

As a practical discipline, the transition from laboratory research to industrial production is essential. However, unlike laboratory research that emphasizes performance enhancement, manufacturing principles prioritize high throughput and cost‐effectiveness. For example, 3D printing enables structural design optimization for electrolytes to achieve remarkable performance [158], but it operates at slow processing speeds, requires stringent rheological properties for slurries, and results in very low production capacity. To enable industrial‐scale manufacturing, proposed methods must satisfy requirements for large‐scale continuous production, maintain costs within reasonable limits, and demonstrate compatibility with diverse formulations. Ideally, these improvements should be directly integrated into existing battery manufacturing processes. Under these stringent conditions, the primary processes that currently meet industrial production requirements include slurry casting, particle spraying, and mixture extrusion.

Slurry casting utilizes liquid solvents as agents to disperse or functionalize solid electrolyte materials and other additives. The well‐blended slurry is deposited onto a substrate, and the film thickness is controlled by adjusting the gap between the blade. As a highly mature and widely adopted approach in electrode manufacturing, extensive existing experience can significantly reduce the initial development barriers for implementing this method in solid electrolyte manufacturing. Additionally, solvent addition reduces interparticle friction, enabling the effective dispersion of precursors and the dissolution of various dispersants and binders, thereby enhancing the uniformity and mechanical properties of solid electrolytes. However, this approach also presents several notable drawbacks. First, it is essential to ensure that the solvents employed do not react with electrolyte precursors or induce material degradation. Second, additional drying processes and solvent recovery steps are required. Considering most organic solvents exhibit some degree of toxicity, necessitating safety measures to protect personnel and the environment during production.

Particle spraying is a coating deposition technique in which particles are accelerated and impacted onto the substrate. Upon impact between the accelerated particles and the substrate, the sprayed particles deposit and bond to the substrate surface. Particle spraying offers superior advantages over other approaches in that it can produce ultra‐thin solid electrolyte films, thereby increasing the overall energy density of solid‐state batteries. Meanwhile, compositional design of particles for individual layers can be achieved through modification of the spraying materials [159]. Despite its numerous benefits, particularly in structural design, the particle spraying process may cause localized overheating and structural damage to electrolyte particles during impact, necessitating significant research efforts to minimize particle degradation and performance deterioration.

Mixture extrusion is a completely solvent‐free manufacturing process that significantly alleviates environmental pollution issues associated with battery manufacturing. Before feeding into the extrusion machine's hopper, particles must be thoroughly blended. The as‐fed mixture undergoes further mixing, plasticization, and homogenization under screw rotation and barrel heating. During this process, bonds between aggregated particles are effectively disrupted, preventing localized material agglomeration. The structure of extruded products is theoretically determined by the machine die; however, their thickness is typically too high for use as electrolytes in high‐energy solid‐state batteries. Therefore, multiple pairs of rollers are currently employed to thin and densify the extruded products [160]. An advanced co‐extrusion process enables enhanced interfacial stability and reduced electrolyte thickness by simultaneously extruding and rolling electrodes and electrolytes [161]. A second challenge that cannot be overlooked is the material selectivity of this process. Materials must possess adequate thermoplasticity and withstand high shear forces without sustaining internal structural damage that would compromise their performance.

Vapor deposition represents a promising method for large‐scale manufacturing that has recently transitioned from laboratory to industrial applications. In this process, materials are dispersed in the gas phase and deposited onto target substrates under specific driving forces. Through this method, solid electrolyte thickness can be precisely controlled to extremely thin dimensions. Through technological iterations and advancements, engineers have recently succeeded in reducing chemical vapor deposition (CVD) manufacturing costs and addressing uniformity issues that arise during scale‐up processes [162]. Consequently, such deposition methods may be widely adopted as viable manufacturing approaches for specialized applications that prioritize ultimate performance over cost efficiency. Given the successful CVD model, numerous laboratory‐stage investigations hold potential for overcoming current challenges and achieving large‐scale industrial manufacturing. The industrializing manufacturing approaches described above each possess distinct advantages and disadvantages. Table 2 outlines key metrics designed to evaluate their characteristics, thereby guiding researchers across various fields to select appropriate methods for fabricating corresponding solid electrolytes.

Following initial electrolyte formation, thermal treatment is essential to heat particles, enhance atomic diffusion, increase product densification, and reduce interfacial resistance for ionic transport. Based on the distinct properties of various electrolyte materials, this process focuses on consolidation while coordinating and controlling temperature, time, pressure, and driving force parameters. This process aims to enhance production capacity, reduce energy consumption, and maintain the electrochemical and mechanical performance of solid electrolytes simultaneously.

### 6.2 Beyond Manufacturing

When developing and exploring optimized manufacturing methods, engineers must select appropriate processing techniques and control the entire manufacturing process. Manufacturing process selection can be broadly categorized into three key aspects: (1) material stability under processing conditions; (2) excellent product performance with high repeatability; and (3) manufacturing processes with high production rates, low costs, simple operations, and minimal environmental impact [163]. After effectively balancing these key considerations, digital control and intelligent manufacturing become essential.

As the core of digital manufacturing, optical systems are employed for quality control and data collection. By adjusting camera configuration, angles, parameters, and image processing algorithms, advanced optical inspection techniques can promptly detect defects in slurries and films, thereby preventing the production of non‐conforming products [164]. Establishing process models based on collected images and battery performance data represents another important direction in digital manufacturing. Researchers anticipate that machine learning can learn and analyze these data, train to identify correlations among different variables, and ultimately provide reliable recommendations to enhance production capacity and optimize processing parameters [165]. However, due to the numerous boundary conditions requiring adjustment and current limitations in computer hardware and software algorithms, further research and optimization remain necessary to achieve highly reliable and robust digital manufacturing systems.

### 6.3 NASICON‐Based Solid Electrolyte Manufacturing

The previous sections have clearly and comprehensively outlined the two key components involved in the fabrication and optimization of NASICON‐based solid electrolytes, namely particle synthesis and film production for ASSB assembly. Further investigation and development toward large‐scale NASICON manufacturing are essential for realizing its commercialization. Building upon the previously evaluated general manufacturing approaches of solid electrolytes, NASICON exhibits inherent strengths and weaknesses, which are discussed in detail below. These unique properties must be carefully considered in large‐scale manufacturing, as they may enable the elimination of certain processing steps or require special process adaptations.

Benefiting from its unique 3D crystalline framework, NASICON exhibits exceptionally high chemical and physical stability. This is exemplified by LATP, which can be stably produced under ambient air conditions [166]. Compared with sulfide‐based solid electrolytes that require strict exclusion of moisture and oxygen, this feature significantly reduces costs associated with production environments, manufacturing equipment, and process development. Additionally, certain NASICON compositions can be processed and operated in water‐based or even strongly alkaline environments [167]. This tolerance to harsh processing conditions further reduces raw material and manufacturing costs. Simultaneously, replacing organic solvents with water during manufacturing alleviates environmental burdens. NASICON is capable of withstanding elevated‐temperature treatment [168], enabling increased drying temperatures and effectively shortening drying times, thereby reducing the footprint of production lines. In addition to its outstanding stability, NASICON is also distinguished by its remarkable mechanical strength, displaying a Young's modulus as high as 97 GPa, a hardness reaching 4.9 GPa, and a fracture strength of up to 110 MPa [169]. These exceptional mechanical performances enable NASICON films to be manufactured at high speeds without concerns regarding mechanical failure or rupture. Moreover, excessive pressure during calendering is less likely to crush NASICON films or degrade their properties. The same superior thermal stability and mechanical strength also hinder effective densification through hot calendering or high‐temperature unidirectional pressing. Low‐density solid electrolytes should be avoided whenever possible, as they not only reduce the battery energy density but also increase the likelihood of dendrite formation and growth, posing potential safety hazards. Beyond these advantages and limitations, manufacturing process optimization is also expected to address one of the most significant challenges associated with related batteries. The extremely high interfacial resistance at the solid electrolyte interfaces severely limits the practical application of NASICON‐based batteries, and the introduction of an additional layer is beneficial. Therefore, this integration of an interfacial protection layer should be explicitly considered in the design of large‐scale manufacturing lines.

## 7 Conclusion and Outlook

In this review paper, we primarily focus on discussing NASICON and its interface with the metal anode, ranging from the fundamental scale to the laboratory scale and, ultimately, the practical scale (i.e., manufacturing). NASICON, in general, has a 3D crystal framework composed of corner‐sharing octahedra and tetrahedra, offering interconnected pathways that facilitate efficient ion transport. This structure can transition between monoclinic and rhombohedral phases, affecting ionic conduction by altering bottleneck geometries along migration pathways. As a result, the Li‐ and Na‐based NASICON materials exhibit ionic conductivities of approximately 10^−4^ S cm^−1^ and 10^−3^ S cm^−1^, respectively.

Despite their favorable ionic conductivity and relatively low cost in the oxide‐solid electrolyte family, NASICON‐type electrolytes face severe interfacial challenges. NASICON is thermodynamically unstable in contact with a metal electrode. The interfacial decomposition products fail to form a passivating layer, thus causing continuous NASICON degradation.

Given the above challenges, we provided a deep understanding of the failure mechanisms at the NASICON‐metal interface from both theoretical and experimental aspects. The failure mechanisms include: (i) Chemical / Electrochemical aspect: the formation of thermodynamically unstable interphases, including SEI layers and secondary phases. These decomposition products often exhibit poor ionic or electronic conductivity, impeding ion transport and increasing interfacial resistance; and (ii) Mechanics aspect: Volume change, stress, and defect buildup, and microstructural imperfections (e.g., voids, and compositional inhomogeneity) at the interface due to the formation of these decomposition products and metal stripping/plating. These factors give rise to cracks, delamination, and metal dendritic growth through the solid electrolyte.

To probe these intricate degradation mechanisms, researchers employ a range of experimental techniques. Spectroscopic methods such as XPS, Raman, TOF‐SIMS, NMR, and XCT provide insight into the chemical composition and evolution of interphases. Electrochemical methods, such as impedance spectroscopy and critical current density tests, quantitatively evaluate interfacial resistance and dendrite tolerance. MOSS and FOS can track the evolution of stress at interfaces. A photoelasticity driven approach allows visualization of stress induced by established dendrites in solid electrolytes. Together, these techniques provide a multi‐dimensional picture of interfacial failure, offering valuable guidance for the rational design of robust NASICON‐based solid‐state battery interfaces.

To experimentally mitigate the interfacial challenges, recent studies have advanced in electrolyte, electrode, and electrolyte‐electrode interface designs. For this review, we covered the popular strategies, including the following: (i) Electrolyte side: doping, artificial secondary phase design, and 3D structure construction, (ii) Metal electrode side: alloying, composite metal, 3D metal host, and stack pressure leading to metal (visco‐)plastic flow, and (iii) Electrolyte ‐electrode interface side: liquid interphase, and solid interlayer, and MIEC designs.

Finally, at a practical implementation level, we reviewed and provided insights into the current state‐of‐the‐art manufacturing approaches applicable to NASICON. These include tape casting, mixture extrusion, and vapor deposition. However, despite the promising progress, manufacturing optimization remains a critical need, particularly in relation to quality control and reproducibility. To address this, we also reviewed the current progress and shared our perspectives on future directions for refining manufacturing processes.

Based on the challenges associated with NASICON and the existing development, we believe the following research directions can be considered in the future NASICON R&D:

1. New NASICON materials chemistry for stable metal interfaces. Significant progress has been achieved through doping strategies and artificial secondary‐phase designs, which have greatly reduced interfacial reactions and suppressed metal dendrite formation. However, current approaches primarily mitigate rather than eliminate these interfacial instabilities. One representative strategy can be using Ta^5+^ substitution. This concept is inspired by Ta‐doped garnet electrolytes, which fully eliminate the solid‐electrolyte/Li‐metal reaction [170, 171]. Systematically to find a class of suitable candidates to stabilize NASICON and its metal interface, a promising route is the integration of physics‐informed machine learning frameworks, trained on DFT‐derived interfacial reaction energies, electrochemical stability windows, and defect energetics, and MD‐predicted ion transport and interfacial mechanical responses, with the relevant high‐throughput experimental synthesis and screening.
2. Optimizing energy density and fast‐charging capability via 3D architectures from both NASICON and metal anode sides. Reduced volumetric energy density caused by incorporating such architectures has been insufficiently addressed. Engineering‐level adjustments are necessary to maximize both energy density and fast‐charging capability. For commercially competitive energy density, the total NASICON thickness must be constrained to lower than 20 µm [172], which is technically achievable through scalable tape‐casting combined with optimized NASICON–ceramic metal host geometries that shorten ion‐transport pathways while preserving dense packing [137, 138, 173]. On the metal anode side, the 3D current‐redistribution function can be realized without introducing additional volume, by engineering the existing current‐collector thickness itself. For example, this can be achieved via chemical etching or patterning of thickness‐fixed metal foils (e.g., Cu) and thus without sacrificing volumetric energy density.
3. Cost‐effectiveness and supply chain considerations. While many advanced NASICON design strategies deliver exceptional cycling performance and are academically impressive, their affordability and scalability are essential for practical deployment. Real‐world applications require careful consideration in terms of material precursor availability, manufacturing costs, and compatibility with existing and future battery supply chains. From a system‐level perspective, a target cost of $50–100 USD kWh^−1^ is necessary to remain competitive with emerging SSBs enabled by NAISCON [172, 174]. Achieving this cost target necessitates electrolyte and cathode chemistries that avoid heavy reliance on rare‐earth or geopolitically constrained elements, while remaining compatible with scalable manufacturing routes such as tape casting, sintering, and roll‐to‐roll processing.
4. Establishing standardized and quantitative evaluation protocols. Despite widespread reporting of critical current density and long‐term symmetric cell cycling performance, there is no consensus on standardized definitions or test protocols. Variations include:
  - Fixed half‐cycle times with incremental current density increases;
  - Fixed half‐cycle times with multiple cycles at each current density;
  - Fixed capacity per half‐cycle with stepwise current density increases;
  - For all three points above, the current density increment size varies between studies.

Similarly, parameters for long‐term symmetric cycling at fixed current density and capacity vary considerably across studies. This lack of uniformity hinders direct comparison between strategies and may slow technological advancement. Establishing community‐wide benchmark protocols is urgently needed.

While community‐wide standardization remains a long‐term goal, a critical near‐term requirement is self–research‐group consistency, in which each group adopts a fixed, transparent testing framework to ensure internal comparability across materials and design iterations. Using consistent protocols within a research group, such as initiating CCD tests at 0.1 mA cm^−2^ with 0.1 mA cm^−2^ current density increments and a fixed half‐cycle capacity of 1.0 mAh cm^−2^, enables meaningful internal comparison across studies [175, 176]. For practical utilization purposes, long‐term symmetric cycling can be conducted at >1 mA cm^−2^ with a fixed areal capacity of 4.0 mAh cm^−2^ [172, 177].

5. Accelerated evaluation via physics‐informed digital twin models. Once standardized testing parameters are established, experimentally determining cycle life can remain a multi‐year endeavor. To shorten development cycles, correlating microstructural and characterization data with electrochemical cycling performance can enable the creation of physics‐informed digital twin models, thereby reducing the need for extensive testing. Key model inputs can include electrolyte porosity, initial interfacial impedance, impedance evolution during repeated cycling, and voltage polarization during formation cycles. These experimentally measurable parameters can be embedded as constraints within physics‐informed machine‐learning frameworks, enabling digital twins trained on early charging/discharging cycles to reliably forecast long‐term interfacial stability and degradation trends.

## Conflicts of Interest

The authors declare no conflicts of interest.

## Data Availability

No original data was used for the research described in the article.
